# Supplementary material for: Photoresponsive spiro-polymers generated in situ by C–H-activated polyspiroannulation
Source: Nat Commun. 2019 Dec 2;10:5483. doi: 10.1038/s41467-019-13308-w (PMC6889291; doi:10.1038/s41467-019-13308-w)
Supplement: Supplementary file 1 — Supplementary Information [file 41467_2019_13308_MOESM1_ESM.pdf]

Supplementary Information

Photoresponsive Spiro-polymers Generated In Situ by C–H-Activated Polyspiroannulation

Han et al.

Supplementary Methods

General information. 2-Naphthol, $Pd(OAc)_2$, $Cu(OAc)_2{\cdot}H_2O$, K_2CO_3 and *N*-methyl-2-pyrrolidon (NMP) were purchased from Nacalai Tesque, Inc., Zhejiang Metallurgical Research Institute Co., Ltd, Advanced Technology & Industrial Company, Fisher Scientific UK and Riedel-de Haen AG, respectively. Dimethyl sulfoxide (DMSO), dimethylformamide (DMF) and NMP used for the model reaction and polymerizations were degassed prior to use. All other reagents and solvents were purchased from Aladdin or Sigma-Aldrich and used without further purification.

Relative number-average (M_n) and weight-average (M_w) molecular weights and polydispersity indices (M_w/M_n) of the polymers were estimated on a Waters gel permeation chromatography (GPC) system equipped with a Waters 486 wavelength-tunable UV-vis detector using THF as an eluent. Details about the sample preparation and the experimental setup can be found in our previous publications.[1-2] Absolute molecular weights were obtained by tandem GPC experiments using a system equipped with an isocratic pump (Waters 2414), a Down HELEOS multi-angle laser light scattering (MALLS) detector and an Optilab rEX refractive index detector (Wyatt technology, Santa Barbara, CA). The separations were carried out using size-exclusion columns (400 Å, 10^3 Å and 10^4 Å phenogel columns, 5 μm, 300 × 7.8 mm, Phenomenex, Torrance, CA) connected in series at 45 ºC with DMF containing 0.1 M LiBr as the mobile phase. The MALLS detector was calibrated by pure toluene without external polymer standards. The absolute M_w of polymers were calculated by their *d*n/*d*c values determined offline using the internal calibration system (ASTRA V software, version 5.1.7.3, Wyatt Technology, Santa Barbara, CA).

FT-IR spectra were recorded on a PerkinElmer 16 PC FTIR spectrophotometer. ^1H and ^{13}C NMR spectra were measured on a Bruker AV 400 NMR spectrometer in CD_2Cl_2 or $CDCl_3$ using the deuterated solvent as the internal standard. High-resolution mass spectra (HRMS) were detected by a MALDI Micro MX mass spectrometer operating in MALDI-TOF mode. The thermogravimetric analyses (TGA) and differential scanning calorimetry (DSC) measurements were conducted using a Perkin-Elmer TGA 7 analyzer and a TA Instruments DSC Q1000, respectively, at a heating rate of 10 °C/min under nitrogen. UV spectra were measured on a PerkinElmer Lambda 365 UV/Vis spectrophotometer. Photoluminescence (PL) spectra were recorded on a Horiba Fluorolog spectrophotometer. Fluorescence quantum yields were measured using a Hamamatsu absolute PL quantum yield spectrometer C11347 Quantaurus QY. The sizes of the nanoaggregates of **7** and P**1a**/**2e** were measured using a Malvern Zetasizer Nano ZSP. The polymer thin films were prepared by spin-coating the 1,2-dichloroethane solutions of polymer samples (~20 mg/mL) on silicon wafers at 1000 rpm for 1 min and then dried in a vacuum oven at room temperature. The polymer solutions were filtered through 0.45 μm PTFE syringe-type filters prior to use. The refractive indices of the polymer films were determined on a J. A. Woollam Variable Angle Ellipsometer with a model of Alpha-SE and a wavelength tunability from 370 to 1690 nm. The 2D photopatterns were generated by UV irradiation of the polymer thin films through a copper photomask for 20 min. The photomask used herein was a grid pattern, in which the square areas were opaque and coated with copper whereas the grid lines were transparent glass substrate. The photo-irradiation process was conducted in air at room temperature using UV light from an Oriel

Mercury Arc Lamp at a distance of 25 cm. The incident light intensity was ~18.5 mW cm^{-2} and the applied power of the Mercury Arc Lamp was 180 W. The photos of the generated photopatterns were taken under normal room light and 330–380 nm UV illumination using a Nikon Eclipse 80i fluorescent microscope.

Monomer synthesis. Monomer **2d** was synthesized by the route as shown in Supplementary Figure 1. 4,4'-(2,2-Diphenylethene-1,1-diyl)bis(bromobenzene) (TPE-2Br) was synthesized according to a previous report.[3] To a 250 mL two-necked round-bottom flask were added $Pd(PPh)_3Cl_2$ (700 mg, 1.0 mmol), CuI (381 mg, 2.0 mmol), PPh_3 (525 mg, 2.0 mmol), TPE-2Br (4.9 g, 10.0 mmol), and a solvent mixture of THF/triethylamine (30 mL/60 mL) under a nitrogen atmosphere. After the solid substrates were completely dissolved, phenylacetylene (4.39 mL, 40.0 mmol) was then injected into the flask through a syringe under stirring and the reaction mixture was heated to 80 °C. After refluxing overnight, the reaction mixture was cooled down to room temperature. The formed solid was removed by filtration and washed with THF for several times. The filtrate was dried by blowing with condensed air and then extracted with dichloromethane. The organic layers were combined and dried over anhydrous sodium sulfate. After filtration and solvent evaporation, the resulting crude product was purified on a silica gel column chromatography using hexane/ethyl acetate as the eluent. Pure product was obtained as a pale yellow solid; yield: 73.5%. ^1H NMR (400 MHz, CD_2Cl_2), δ (ppm): 7.54–7.52 (m, 4H), 7.39–7.28 (m, 10H), 7.17–7.15 (m, 6H), 7.10–7.04 (m, 8H). ^{13}C NMR (100 MHz, CD_2Cl_2), δ (ppm): 143.71, 143.39, 142.50, 139.74, 131.70, 131.57, 131.45, 131.19, 128.47, 128.36, 128.00, 126.98, 123.41, 121.42, 89.90, 89.63. HRMS (MALDI-TOF, *m*/*z*): M^+ calcd. for $C_{42}H_{28}$, 532.2191; found 532.2180.

Characterization data of polymers. ***P1a/2a***: Light yellow solid; yield: 99% (Table 1, entry 5). M_n: 12, 200; M_w: 21,300; M_w/M_n: 1.8 (GPC, polystyrene calibration). IR (KBr), v (cm^{-1}): 3053, 2938, 2864, 1661, 1604, 1508, 1393, 1284, 1242, 1176, 1018, 833, 761, 698. ^1H NMR (400 MHz, CD_2Cl_2), δ (ppm): 7.37–7.30, 7.15, 7.02–6.94, 6.91–6.87, 6.64, 6.53, 6.46, 6.16, 3.84, 3.76, 1.70, 1.43. ^{13}C NMR (100 MHz, CD_2Cl_2), δ (ppm): 196.23, 158.62, 158.43, 148.29, 147.95, 147.69, 147.34, 147.27, 146.68, 146.60, 146.05, 139.25, 139.13, 139.01, 136.13, 135.38, 133.34, 131.61–127.09, 113.95, 76.16, 68.06, 29.53, 26.20.

P1a/2b: Light yellow solid; yield: 95%. M_n: 12,700; M_w: 24,400; M_w/M_n: 1.9 (GPC, polystyrene calibration). IR (KBr), v (cm^{-1}): 3051, 2946, 2872, 1660, 1604, 1508, 1393, 1284, 1242, 1176, 1018, 833, 761, 698. ^1H NMR (400 MHz, $CDCl_3$), δ (ppm): 7.32–7.22, 7.09, 7.00–6.84, 6.64–6.51, 6.41, 6.16, 3.86, 3.77, 1.80. ^{13}C NMR (100 MHz, $CDCl_3$), δ (ppm): 196.33, 157.96, 157.77, 147.74, 147.45, 147.22, 146.94, 146.83, 146.77, 146.33, 146.28, 145.77, 139.11, 139.00, 138.88, 135.65, 135.00, 131.70–126.75, 113.75, 75.93, 67.15, 26.06.

P1a/2c: Light yellow solid; yield: 42%. M_n: 9,700; M_w: 14,300; M_w/M_n: 1.5 (GPC, polystyrene calibration). IR (KBr), v (cm^{-1}): 3053, 2929, 2853, 1661, 1604, 1509, 1393, 1285, 1243, 1175, 1020, 832, 761, 697. ^1H NMR (400 MHz, $CDCl_3$), δ (ppm): 7.32–7.24, 7.11, 7.02–6.85, 6.65–6.59, 6.53, 6.44, 6.17, 3.83, 3.74, 1.71, 1.64, 1.27. ^{13}C NMR (100 MHz, $CDCl_3$), δ (ppm): 196.38, 158.19, 158.01, 147.80, 147.51, 147.18, 146.90, 146.34, 146.27, 145.73, 139.23, 139.11, 135.78, 135.73, 135.09, 133.17, 131.58–126.73, 113.79, 75.97, 67.86, 29.58, 29.46, 29.37, 26.14.

P1a/2d: Yellow solid; yield: 87%. M_n: 5,100; M_w: 7,300; M_w/M_n: 1.5 (GPC, polystyrene calibration). IR (KBr), ν (cm^{-1}): 3054, 3025, 1664, 1597, 1566, 1493, 1442, 1397, 1235, 1200, 1110, 1071, 1024, 841, 758, 697. ^{1}H NMR (400 MHz, $CDCl_3$), δ (ppm): 7.31–6.26, 6.13. ^{13}C NMR (100 MHz, $CDCl_3$), δ (ppm): 195.95, 148.01, 147.80, 147.22, 146.85, 145.69, 143.79, 143.61, 143.48, 142.33, 141.92, 140.91, 140.65, 140.49, 138.69, 135.28, 135.03, 134.60, 133.49, 132.68, 131.66–126.07, 123.43, 75.93.

P1a/2e: Yellow solid; yield: 87%. M_n: 5,700; M_w: 9,900; M_w/M_n: 1.7 (GPC, polystyrene calibration). IR (KBr), ν (cm^{-1}): 3055, 3027, 1664, 1597, 1566, 1494, 1443, 1397, 1235, 1199, 1111, 1072, 1023, 843, 758, 697. ^{1}H NMR (400 MHz, $CDCl_3$), δ (ppm): 7.31–6.27, 6.13. ^{13}C NMR (100 MHz, $CDCl_3$), δ (ppm): 195.87, 148.01, 147.73, 147.28, 146.98, 145.78, 143.68, 143.35, 142.18, 141.36, 141.06, 140.70, 138.71, 135.33, 134.64, 132.86, 131.69–126.50, 123.43, 75.97.

P1b/2a: Light yellow solid; yield: 95%. M_n: 5,400; M_w: 10,300; M_w/M_n: 1.9 (GPC, polystyrene calibration). IR (KBr), ν (cm^{-1}): 3051, 2938, 2865, 1665, 1604, 1570, 1508, 1470, 1445, 1385, 1285, 1245, 1177, 1109, 1019, 834, 760, 699. ^{1}H NMR (400 MHz, $CDCl_3$), δ (ppm): 8.54, 7.58–7.43, 7.31–7.22, 7.12, 6.99, 6.85, 6.62, 6.51–6.41, 3.95, 3.85, 3.77, 1.71, 1.46. ^{13}C NMR (100 MHz, $CDCl_3$), δ (ppm): 195.38, 159.27, 158.28, 158.17, 149.37, 149.12, 147.52, 146.94, 146.58, 145.87, 135.24, 134.56, 133.16, 131.57, 131.46, 131.39, 130.21, 129.12, 128.43, 128.03, 127.14, 126.51, 124.36, 123.76, 115.28, 114.65, 114.02, 113.90, 75.21, 68.00, 29.25, 26.00.

Model reaction. A 50 mL Schlenk tube equipped with a stirring bar was charged with 2-naphthol (**1a**, 288.3 mg, 2.0 mmol), diphenylacetylene (**3**, 2.23 g, 5.0 mmol), $Pd(OAc)_2$ (22.5 mg, 0.1 mmol), $Cu(OAc)_2{\cdot}H_2O$ (838.5 mg, 4.2 mmol), and K_2CO_3 (552.8 mg, 4.0 mmol), and then sealed with a rubber stopper. After evacuated under vacuum and purged with dry nitrogen for three times, the tube was injected with 20 mL DMSO. The reaction mixture was heated at 120 °C for 48 h, and then cooled down to room temperature. Water was then added to the mixture, followed by sequential extraction with ethyl acetate and drying over anhydrous $MgSO_4$. After solvent evaporation, the crude product was subjected to a silica-gel column using hexane/ethyl acetate mixture (10:1, v/v) as eluent. The desired model compound **4** was collected as a yellow solid; yield: 20.1%. IR (KBr), υ (cm^{-1}): 3051, 3025, 1659, 1621, 1596, 1565, 1489, 1442, 1393, 1233, 1199, 1073, 1029, 844, 808, 764, 731, 699. ^{1}H NMR (400 MHz, CD_2Cl_2), δ (ppm): 7.40–7.31 (m, 5H), 7.18–7.11 (m, 6H), 7.04–7.00 (m, 6H), 6.97–6.93 (m, 4H), 6.64 (d, J = 8.0 Hz, 4H), 6.16 (d, J = 8.0 Hz, 1H). ^{13}C NMR (100 MHz, CD_2Cl_2), δ (ppm): 195.83, 148.52, 147.65, 146.11, 138.64, 135.79, 135.05, 131.09, 130.91, 130.41, 130.18, 129.47, 128.22, 128.10, 127.69, 127.44, 127.41, 127.34, 76.38. HRMS (MALDI-TOF, *m*/*z*): $[M+H]^+$ calcd. for $C_{38}H_{27}O$, 499.2062; found 499.2073.

Through a similar experiment procedure, model compound **6** was prepared by the oxidative coupling of 2-naphthol (43.3 mg, 0.3 mmol) and methoxyl-substituted internal alkyne **5** (250.0 mg, 1.2 mmol) in the presence of $Pd(OAc)_2$ (6.7 mg, 0.03 mmol), $Cu(OAc)_2{\cdot}H_2O$ (125.8 mg, 0.63 mmol), and K_2CO_3 (82.9 mg, 0.6 mmol) in DMSO (3 mL) at 120 ℃ for 48 h. A mixture of three isomers (**6a–c**) was collected as a yellow solid, which was hard to be separated by column chromatography because of their similar physical properties. Yield: 41.6%. ^{1}H NMR (400 MHz, $CDCl_3$), δ (ppm): 7.34–7.24 (m, 6H), 7.14–7.10 (m, 2H), 7.03–6.88 (m, 7H),

6.66–6.62 (m, 4H), 6.55 (d, J = 8.0 Hz, 2H), 6.47 (d, J = 8.0 Hz, 2H), 6.20–6.16 (m, 1H), 3.74–3.64 (m, 6H). ^{13}C NMR (100 MHz, $CDCl_3$), δ (ppm): 196.38, 158.53, 158.36, 150.60, 149.13, 147.68, 147.01, 146.75, 146.39, 145.81, 138.99, 135.67, 134.99, 133.79, 132.32, 131.99, 131.50, 131.43, 130.65, 130.59, 130.33, 130.08, 129.71, 129.25, 128.75, 128.65, 128.32, 128.23, 127.93, 127.87, 127.79, 127.59, 127.47, 127.20, 127.10, 126.91, 126.76, 124.79, 124.33, 113.83, 113.33, 113.27, 75.93, 55.49, 55.14, 55.05. HRMS (MALDI-TOF, *m*/*z*): M^+ calcd. for $C_{40}H_{30}O_3$, 558.2195; found 558.2182.

Analysis of the filtrate composition. To investigate what happened for the excess **1a**, the filtrate was purified and analyzed after the filtration treatment process of the polymerization between 2-naphthol (173.0 mg, 1.2 mmol) and internal diyne **2a** (141.2 mg, 0.3 mmol). Two pure compounds were isolated from the precipitant mixture by evaporation of the solvents in vacuo and the subsequent column chromatography on silica gel using DCM as eluent. The structures of the isolated products were verified to be 2-naphthol and 1,1-bi-2naphthol, respectively, by HRMS, ^{1}H NMR and ^{13}C NMR (Supplementary Figures 4–11). The recovery yield of 2-naphthol (59.0 mg) was calculated to be 46% and the yield of 1,1-bi-2naphthol (11.7 mg) was determined to be 9%.

Preparation of telechelic polymer. Into a 10 mL Schlenk tube with a stirring bar was added 2-naphthol (57.7 mg, 0.4 mmol), internal diyne **2a** (188.2 mg, 0.4 mmol), $Pd(OAc)_2$ (18.0 mg, 0.08 mmol), $Cu(OAc)_2 \cdot H_2O$ (335.4 mg, 0.84 mmol), and K_2CO_3 (221.2 mg, 1.6 mmol) in 2 mL DMSO. The reaction mixture was stirred under nitrogen at 120 ℃ for 24 h and then cooled to room temperature. After the same precipitation procedures as that of the aforementioned polymerizations, the precipitates were collected by filtration, and then washed with hexane and dried in vacuum at room temperature to a constant weight. A light yellow solid of P**1a**/**2a** oligomer was obtained. M_n = 3,800; M_w = 5,900; M_w/M_n = 1.6 (GPC, polystyrene calibration).

Polymerization of telechelic polymer. Into a 10 mL Schlenk tube equipped with a stirring bar was placed with excess 2-naphthol (45.3 mg), telechelic polymer P**1a**/**2a** (55 mg, 14.5 μmol), $Pd(OAc)_2$ (3.5 mg, 2.9 μmol), $Cu(OAc)_2 \cdot H_2O$ (65.9 mg, 60.9 μmol), and K_2CO_3 (43.4 mg, 58.0 μmol) in 1 mL DMSO. The reaction mixture was stirred under nitrogen at 120 ℃ for 24 h and then cooled to room temperature. The resulting mixture was first dissolved with THF and centrifuged for several times. Then the supernatant solution was passed through a simple column filled with neutral Al_2O_3 powder and added dropwise to 160-mL hexane/chloroform mixture (7:1 v/v) under vigorous stirring. The precipitates were collected by filtration, and then washed with hexane and dried in vacuum at room temperature to a constant weight. A light yellow solid of extended P**1a**/**2a** was obtained in a yield of 95%. M_n = 9,600; M_w = 24,100; M_w/M_n = 2.5 (GPC, polystyrene calibration). Polymer P**1ac**/**2a** and P**1ad**/**2a** was prepared in similar procedures by using excess 6-methoxyl-2-naphthol (54.7 mg) and 6-benzoyl-2-naphthol (78.0 mg) as the co-monomer to react with telechelic polymer P**1a**/**2a** (55.0 mg). The characterization data of P**1ac**/**2a** and P**1ad**/**2a** were given as follows:

P1ac/2a: Light yellow solid; yield: 76%. M_n: 10,400; M_w: 23,400; M_w/M_n: 2.3 (GPC, polystyrene calibration). IR (KBr), ν (cm^{-1}): 3054, 2939, 2863, 1660, 1604, 1508, 1392, 1283, 1241, 1176, 1027, 833, 763, 698. ^{1}H NMR (400 MHz, $CDCl_3$), δ (ppm): 7.37–7.19, 7.11,

7.01–6.86, 6.66–6.43, 6.17, 3.89, 3.84, 3.75, 1.70, 1.43. ^{13}C NMR (100 MHz, $CDCl_3$), δ (ppm): 196.36, 158.09, 157.91, 147.77, 147.48, 147.20, 146.91, 146.81, 146.32, 146.25, 145.75, 139.17, 139.05, 135.73, 135.68, 135.04, 131.72–126.26, 113.76, 75.94, 67.70, 67.57, 55.44, 29.30, 25.99.

P1ad/2a: Light yellow solid; yield: 92%. M_n: 13,200; M_w: 29,600; M_w/M_n: 2.2 (GPC, polystyrene calibration). IR (KBr), ν (cm^{-1}): 3055, 2938, 2863, 1659, 1610, 1508, 1392, 1284, 1242, 1177, 1017, 834, 766, 699. ^{1}H NMR (400 MHz, $CDCl_3$), δ (ppm): 7.85–7.72, 7.61–7.44, 7.32, 7.24, 7.10, 7.00–6.84, 6.64–6.41, 6.17, 3.82, 3.74, 1.65, 1.42. ^{13}C NMR (100 MHz, $CDCl_3$), δ (ppm): 196.47, 195.67, 158.09, 157.91, 147.74, 147.75, 147.18, 146.90, 146.83, 146.34, 146.27, 145.83, 139.05, 137.34, 136.68, 135.72, 135.67, 135.03, 132.81, 131.47–126.26, 113.76, 75.92, 68.10, 67.63, 29.30, 25.98, 25.74.

Reduction reaction of 4. A 100 mL, two-necked, round-bottomed flask fitted with a stirring bar was charged with 10 mL of anhydrous THF and 20 mg $LiAlH_4$ (0.5 mmol) under a nitrogen atmosphere at an ice-water bath. Model compound **4** (200 mg, 0.4 mmol) in anhydrous THF (2 mL) was then added dropwise via a syringe to the cooled suspension of $LiAlH_4$. The mixture was kept stirring at 0 °C. The reaction was completed after about 30 min as monitored by the thin-layer chromatography. Afterward, the reaction was quenched by dropwise addition of saturated NH_4Cl aqueous solution. The mixture was extracted with DCM three times and the organic layers were combined and dried over anhydrous Na_2SO_4. The solvent was then removed in vacuo, leaving 180 mg of **34** (90.0% yield) as a pale yellow solid. IR (KBr), υ (cm^{-1}): 3053, 3023, 2920, 2850, 1707, 1596, 1574, 1486, 1440, 1412, 1223, 1158, 1071, 1026, 916, 848, 806, 769, 741, 696. ^{1}H NMR (400 MHz, CD_2Cl_2), δ (ppm): 7.36–7.34 (m, 1H), 7.24–7.16 (m, 2H), 7.12–6.95 (m, 17H), 6.75–6.72 (m, 4H), 2.72 (t, J = 8.0 Hz, 2H), 2.40 (t, J = 8.0 Hz, 2H). ^{13}C NMR (100 MHz, CD_2Cl_2), δ (ppm): 208.27, 149.64, 145.94, 136.76, 135.58, 135.08, 134.29, 130.26, 129.71, 128.67, 127.94, 127.70, 127.40, 127.29, 127.25, 127.05, 126.91, 77.77, 40.69, 28.02. HRMS (MALDI-TOF, m/z): M^+ calcd. for $C_{38}H_{28}O$, 500.2140; found 500.2141.

Reduction reaction of P1a/2a. A 100 mL, two-necked, round-bottomed flask fitted with a stirring bar was charged with 10 mL of anhydrous THF and 10 mg $LiAlH_4$ (0.26 mmol) under a nitrogen atmosphere at an ice-water bath. P**1a**/**2a** (92.7 mg, 0.15 mmol) in anhydrous THF (3 mL) was then added dropwise via a syringe to the cooled suspension of $LiAlH_4$. The mixture was kept stirring at 0 °C. The reaction was stopped after about 30 min. Afterward, the reaction was quenched by dropwise addition of saturated NH_4Cl aqueous solution. The mixture was extracted with DCM three times and the organic layers were combined and dried over anhydrous Na_2SO_4. The solvent was then removed in vacuo to give the reduced product P**7**.

Characterization Data for P**7**: Pale yellow solid; yield: 89%. M_n: 8,700; M_w: 18,000; M_w/M_n: 2.1 (GPC, polystyrene calibration). The GPC data of the P**1a**/**2a** reactant is: M_n = 8,800, M_w = 18,500, and M_w/M_n = 2.1. IR, υ (cm^{-1}): 3054, 3025, 2927, 2856, 1712, 1604, 1574, 1507, 1490, 1471, 1442, 1411, 1392, 1346, 1282, 1238, 1174, 1069, 1010, 926, 831, 804, 765, 745, 696. ^{1}H NMR (400 MHz, CD_2Cl_2), δ (ppm): 7.31–7.14, 7.07–6.96, 6.85–6.81, 6.73–6.71, 6.62–6.49, 3.81–3.76, 2.72, 2.39, 1.69, 1.42. ^{13}C NMR (100 MHz, CD_2Cl_2), δ (ppm): 208.41, 158.53,

149.85, 149.58, 149.19, 148.94, 146.01, 145.70, 137.17, 136.28, 135.95, 135.02, 134.93, 133.37, 131.95–126.20, 114.12, 113.93, 77.91, 68.02, 40.90, 29.57, 28.37, 26.21.

Photonic device experiments and simulations. The Si_3N_4 devices used in this work were fabricated on a silicon chip in the Nanosystem Fabrication Facility (NFF) of The Hong Kong University of Science and Technology. We deposited a Si_3N_4 film by low-pressure chemical-vapor deposition (LPCVD) in two consecutive runs with a total thickness of ~0.82 μm on an oxide layer of ~4 mm thick. We measured the refractive index of the Si_3N_4 film to be ~1.96 at 1550 nm wavelength using ellipsometry. We defined the device pattern on the Si_3N_4 layer by i-line (365 nm) photolithography and inductively coupled plasma (ICP) etching (STS ICP DRIE silicon etcher). The polymer film of P**1a**/**2a** was spin-coated on the devices at a spin speed of 1000 r/min for 1 min.

We designed the six racetrack microring resonators under test with the same round-trip lengths of 190π μm but different waveguide-resonator coupling gap widths (D1–D3: 0.4 μm; D4–D6: 0.5 μm) and coupling lengths (D1, D6: 9.4 μm; D2, D5: 6.3 μm; D3, D4: 3.1 μm). The photo-irradiation process was conducted in air at room temperature using UV light from an Oriel Mercury Arc Lamp at a distance of 25 cm. The incident light intensity was ∼18.5 mW cm^{-2} and the applied power of the Mercury Arc Lamp was 180 W. For transmission spectra measurements, we employed a wavelength-tunable laser (Santec TSL-510) in the 1550 nm wavelength range. The laser was operated at room temperature. We used a lensed polarization-maintaining single-mode optical fiber to input-couple light by end-firing into the waveguide tapered end-face of ~4 mm width. The optical power from the lensed fiber output before coupling to the chip was ~1 mW. We used a long-working-distance microscope objective lens with a numerical aperture of 0.55 to output-couple light from the chip. Typical insertion loss was 10–15 dB depending on the alignment. The results and analysis for TE polarization are shown in Supplementary Figures 54–57.

We employed finite-element method (FEM) to calculate the effective refractive index of polymer-coated Si_3N_4 waveguides using the commercial modeling software COMSOL Multiphysics. We adopted the Si_3N_4 waveguide core and the silica bottom-cladding layer with refractive indices of 1.96 and 1.44, respectively. We adopted the waveguide width and height to be 1.2 μm and 0.82 μm, respectively. We assumed a uniform polymer thickness on the waveguide top and side surfaces for simplicity, although the polymer thickness on the waveguide side surfaces are typically thinner in practice. The resonance wavelength of a microring is determined by the phase-matching condition: $n_{eff}L = m\lambda_m$, where L is the round-trip length of the microring, m is the integral mode number, and λ_m is the m-th order resonance wavelength in a vacuum. Therefore, the resonance wavelength λ_{res} is sensitive to n_{eff}.

In order to better correlate the observed blue-shift ($\Delta\lambda$) of resonance wavelength to the UV-induced polymer refractive index change (Δn), we prepared several polymer films coated on unpatterned silicon chips using the same spin-coating conditions as those coated on the microring devices. Then we measured the refractive index of these polymer films on unpatterned silicon chips upon different UV exposure durations. The corresponding change in the n value at 1550 nm is shown in Figure 7g.

The measured $|\Delta\lambda/\lambda_{res}|$ was compared with the FEM-simulated results, where we use the measured n and Δn of the polymer film to calculate the n_{eff} and Δn_{eff} and assume that $\Delta\lambda/\lambda_{res}$ =

$\Delta n_{eff}/n_{eff}$ by following the phase-matching condition. The polymer thickness coated on the devices was estimated by finding the closest match between the experimental data of $|\Delta\lambda/\lambda_{res}|$ of the six devices and the simulation results with different polymer thickness. For each device, two estimated polymer thickness values are obtained based on TM and TE polarizations. The estimation based on TM and TE polarization is sensitive to the polymer thickness on the waveguide top surface and the waveguide sidewall, respectively.

Supplementary Tables

Supplementary Table 1. Effect of catalyst on the polymerization of 1a and 2a[a]

entry	catalyst	catalyst loading (equiv.)	yield (%)	M_n[b]	M_w[b]	$Đ$[b]
1	$Pd(OAc)_2$	0.10	25	3600	4300	1.2
2	$Pd(dppf)Cl_2 \cdot CH_2Cl_2$	0.10	27	3400	3900	1.2
3	$Pd(PPh_3)_2Cl_2$	0.10	30	3600	4300	1.2
4	$Pd(OAc)_2$	0.05	4	3100	3300	1.1
5	$Pd(OAc)_2$	0.20	44	4100	5400	1.4

[a] Carried out in DMSO under nitrogen at 120 °C for 24 h in the presence of palladium catalyst, $Cu(OAc)_2 \cdot H_2O$ and K_2CO_3. [**1a**] = [**2a**] = 0.20 M, [Cu] = 0.84 M, [K_2CO_3] = 0.80 M. [b] Determined by GPC in THF on the basis of a linear polystyrene calibration. $Đ$ = polydispersity = M_w/M_n.

Supplementary Table 2. Effect of solvent and temperature on the polymerization of 1a and 2a[a]

entry	solvent	temp (°C)	yield (%)	M_n[b]	M_w[b]	$Đ$[b]
1[c]	DMSO	120	44	4,100	5,400	1.4
2[d]	DMSO/H_2O[d]	120	6	3,200	3,700	1.2
3	DMF	100	14	3,400	3,800	1.1
4	NMP	120	31	3,000	4,000	1.3
5	*o*-xylene	140	7	3,500	4,300	1.2

[a] Carried out under nitrogen at 120 °C for 24 h in the presence of $Pd(OAc)_2$, $Cu(OAc)_2 \cdot H_2O$ and K_2CO_3. [**1a**] = [**2a**] = 0.20 M, [Pd] = 0.04 M, [Cu] = 0.84 M, [K_2CO_3] = 0.80 M. DMSO = Dimethyl sulfoxide; DMF = dimethylformamide; NMP=*N*-methyl-2-pyrrolidone. [b] Determined by GPC in THF on the basis of a linear polystyrene calibration. $Đ$ = polydispersity = M_w/M_n. [c] Data taken from Supplementary Table 1, entry 5. [d] 4:1 by volume.

Supplementary Table 3. Effect of additive and oxidant loading on the polymerization of 1a and 2a[a]

entry	[K_2CO_3] (M)	[$Cu(OAc)_2 \cdot H_2O$] (M)	yield (%)	M_n[b]	M_w[b]	M_w/M_n[b]
1	0	0.84	69	4,800	6,500	1.4
2[c]	0.80	0.84	99	12,200	21,300	1.8
3	0.80	0.42	30	3,700	4,500	1.2
4	0.80	1.00	93	9,000	16,800	1.9

[a] Carried out in DMSO under nitrogen at 120 °C for 24 h in the presence of $Pd(OAc)_2$, $Cu(OAc)_2 \cdot H_2O$. [**1a**] = 0.80 M, [**2a**] = 0.20 M, [Pd] = 0.04 M. [b] Determined by GPC in THF on the basis of a linear polystyrene calibration. $Đ$ = polydispersity = M_w/M_n. [c] Data taken from Table 1, entry 5.

Supplementary Table 4. Effect of monomer concentration on the polymerization of 1a and 2a[a]

entry	[**2a**] (M)	yield (%)	M_n[b]	M_w[b]	M_w/M_n[b]
1	0.10	73	4,900	6,800	1.4
2[c]	0.20	99	12,200	21,300	1.8
3	0.40	83	12,400	22,100	1.8

[a] Carried out in DMSO under nitrogen at 120 °C for 24 h in the presence of $Pd(OAc)_2$, $Cu(OAc)_2 \cdot H_2O$ and K_2CO_3. [**1a**] = 4[**2a**], [Pd] = 0.2[**2a**], [Cu] = 4.2[**2a**], [K_2CO_3] = 4[**2a**]. [b] Determined by GPC in THF on the basis of a linear polystyrene calibration. *Đ* = polydispersity = M_w/M_n. [c] Data taken from Table 1, entry 5.

Supplementary Table 5. Time course of the polymerization of 1a and 2a[a]

entry	time (h)	yield (%)	M_n[b]	M_w[b]	M_w/M_n[b]
1	0.25	76	9,500	21,000	2.2
2	0.75	87	9,600	22,500	2.4
3	1	88	9,900	20,700	2.1
4	3	89	11,200	25,300	2.3
5	6	92	10,500	23,200	2.2
6	12	96	10,800	20,800	1.9
7	18	96	11,000	19,700	1.8
8[c]	24	99	12,200	21,300	1.8

[a] Carried out in DMSO under nitrogen at 120 °C for 24 h in the presence of $Pd(OAc)_2$, $Cu(OAc)_2 \cdot H_2O$ and K_2CO_3. [**1**] = 0.80 M, [**2**] = 0.20 M, [Pd] = 0.04 M, [Cu] = 0.84 M, [K_2CO_3] = 0.80 M. [b] Determined by GPC in THF on the basis of a linear polystyrene calibration. [c] Data taken from Table 1, entry 5.

Supplementary Table 6. Refractive indices and chromatic dispersions of P1/2[a]

no.	polymer	t (min)	$n_{632.8}$	n_{1550}	ν_D	ν_D'	D	D'
1	**P1a/2a**	0	1.6526	1.6158	13.4	93.1	0.075	0.011
2	**P1a/2b**	0	1.6649	1.6338	13.9	129.7	0.072	0.008
3	**P1a/2c**	0	1.6407	1.5733	11.8	36.0	0.085	0.028
4	**P1a/2d**	0	1.6820	1.6288	10.0	64.5	0.100	0.016
5	**P1a/2e**	0	1.6896	1.6468	10.1	101.0	0.099	0.010
6	**P1b/2a**	0	1.6739	1.6311	14.2	72.5	0.071	0.014
7	**P1a/2a**	10	1.6337	1.6011	16.9	91.9	0.059	0.011
8	**P1a/2a**	20	1.6310	1.5913	15.9	69.2	0.063	0.014
9	**P1a/2a**	30	1.6232	1.5885	17.5	79.6	0.057	0.013
10	**P1a/2a**	40	1.6090	1.5790	17.5	97.2	0.057	0.010
11	**P7**	0	1.6320	1.6021	19.2	99.6	0.052	0.010

[a] Samples taken from Table 1, entries 6–11. Abbreviation: t = UV irradiation time, n = refractive index, ν_D = Abbé number = $(n_D - 1)/(n_F - n_C)$, where n_D, n_F, and n_C are the n values at wavelengths of Fraunhofer D, F, and C spectral lines of 589.2, 486.1, and 656.3 nm, respectively; ν_D' = modified Abbé number = $(n_{1319} - 1)/(n_{1064} - n_{1550})$, where n_{1319}, n_{1064}, and n_{1550} are the n values at 1319, 1064, and 1550 nm, respectively. The wavelengths of 1319 and 1064 nm are chosen in view of the practical interest of a commercial laser wavelength (Nd:YAG), while 1550 nm is chosen due to its telecommunication importance. D = chromatic dispersion in the visible region = $1/\nu_D$. D' = chromatic dispersion in the IR region = $1/\nu_D'$.

Supplementary Table 7. Estimation results of polymer thickness coated on the devices[a]

	D1	D2	D3	D4	D5	D6
Estimation based on TM (nm)	90	80	90	60	80	100
Estimation based on TE (nm)	70	60	60	40	60	70
Estimated thickness of device (nm)	70–90	60–80	60–90	50–70	60–80	70–100

[a] The polymer thickness coated on the devices was estimated by finding the closest match between the experimental data of the six devices and the FEM-simulated results with different polymer thickness. The measured $|\Delta\lambda/\lambda_{res}|$ is compared with the simulation results, where the measured n and Δn of the polymer film is used to calculate the n_{eff} and Δn_{eff} and $\Delta\lambda/\lambda_{res} = \Delta n_{eff}/n_{eff}$ is assumed according to the phase-matching condition. For each device, the estimated value from TM and TE polarization is set as the upper and lower limit, respectively. The results indicate that the uncertainty for the estimated polymer thickness is 30 nm.

Supplementary Figures

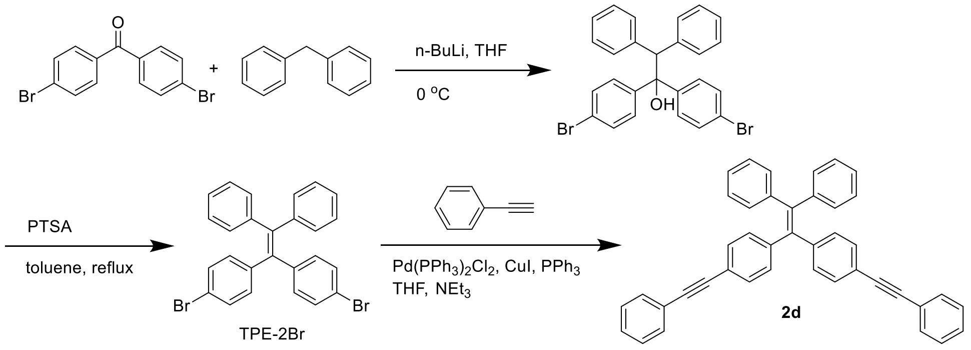

Supplementary Figure 1. Synthetic routes to monomer **2d**.

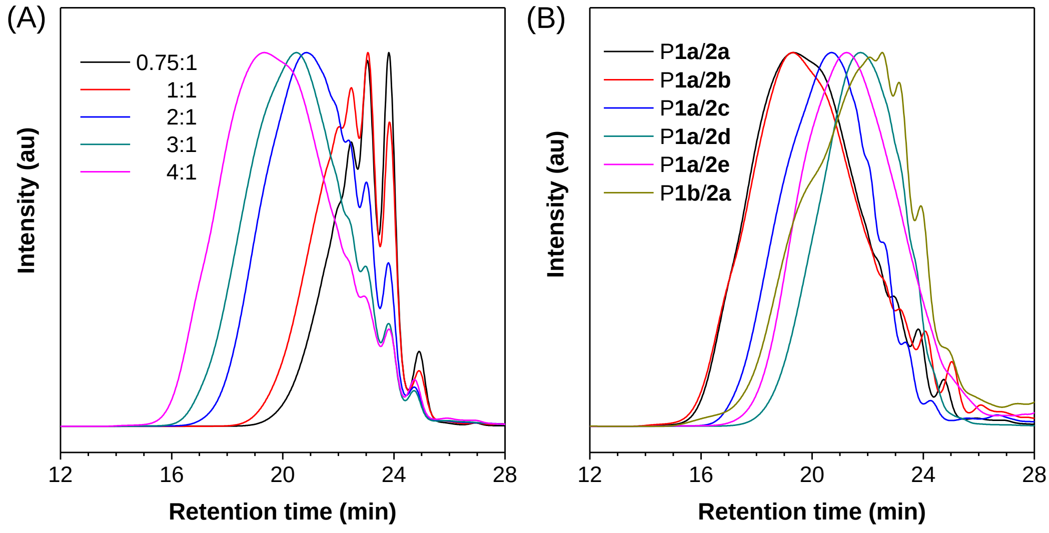

Supplementary Figure 2. Overlay of the GPC traces of the polymers reported in (A) Table 1, entries 1–5 and (B) Table 1, entries 5–10 (measured by GPC in THF on the basis of a linear polystyrene calibration).

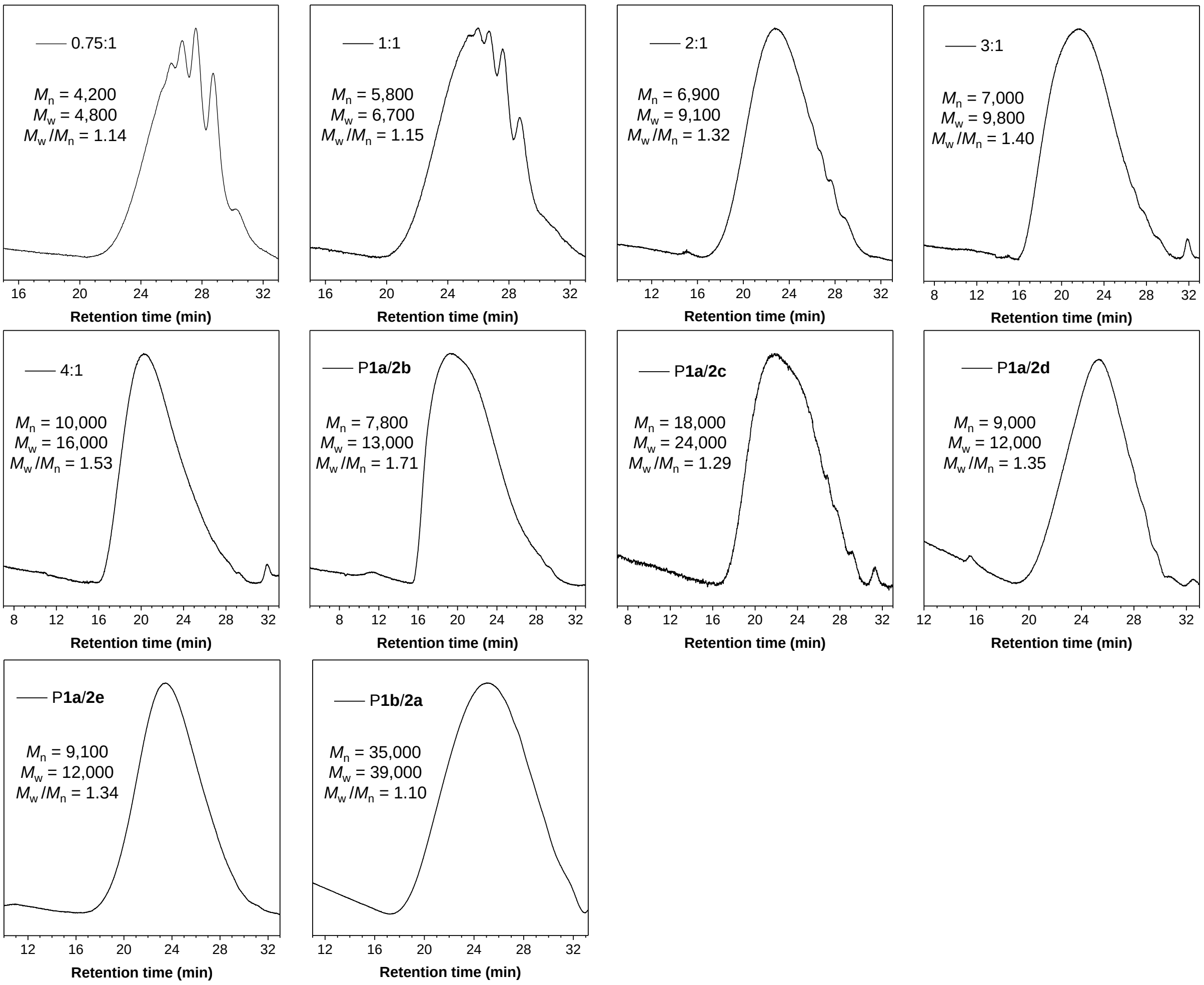

Supplementary Figure 3. GPC traces of the polymers reported in Table 1 (measured in DMF containing 0.1 M LiBr using a MALLS detector).

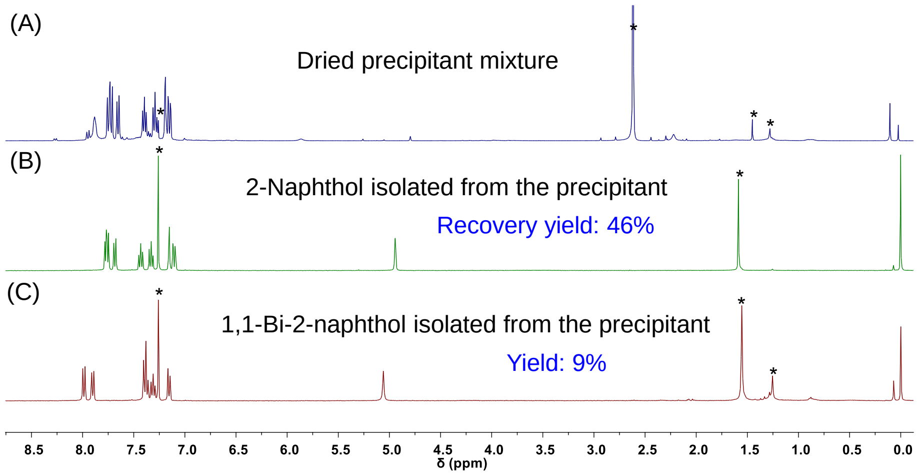

Supplementary Figure 4. Comparison of the ^{1}H NMR spectra of the dried precipitant mixture and the isolated products in $CDCl_3$.

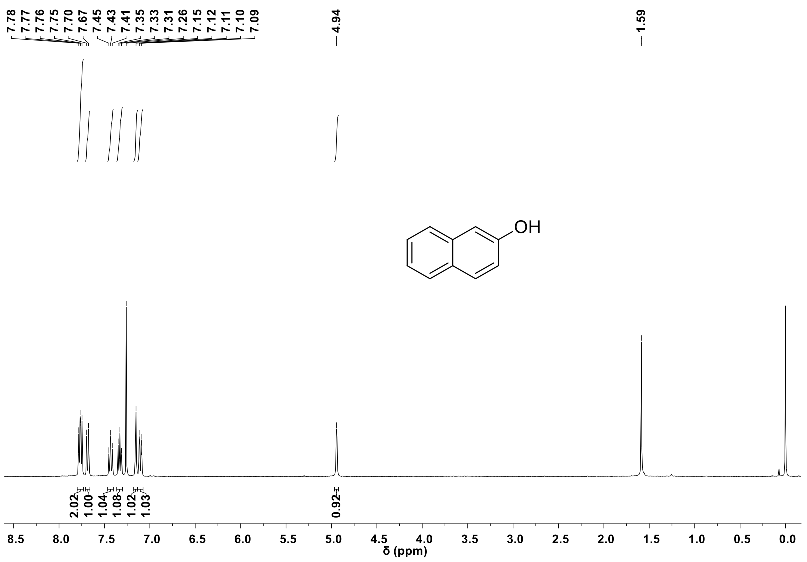

Supplementary Figure 5. ^{1}H NMR spectra of the 2-naphthol product isolated from the precipitant in $CDCl_3$.

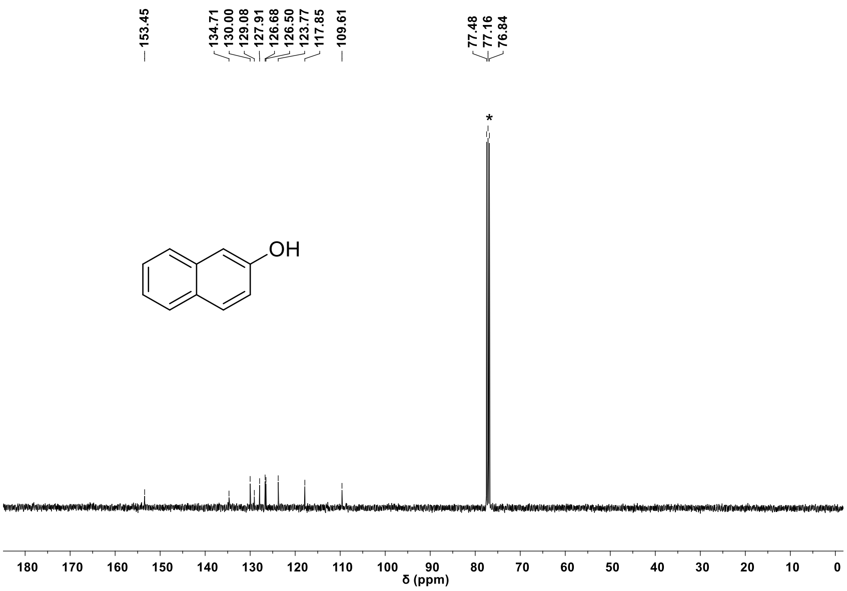

Supplementary Figure 6. ^{13}C NMR spectra of the 2-naphthol product isolated from the precipitant in $CDCl_3$.

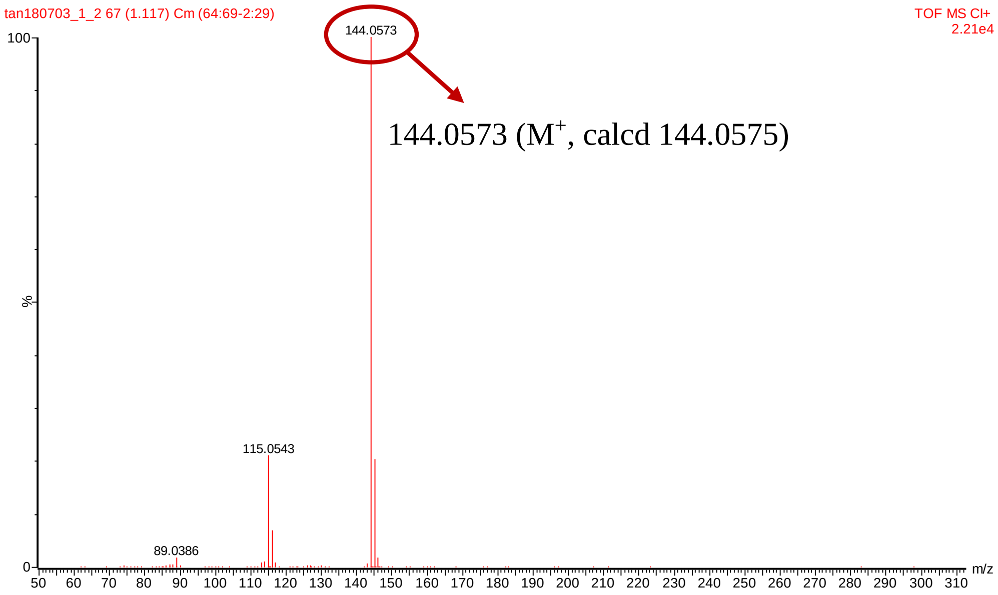

Supplementary Figure 7. HRMS (MALDI-TOF) spectrum of the 2-naphthol product isolated from the precipitant.

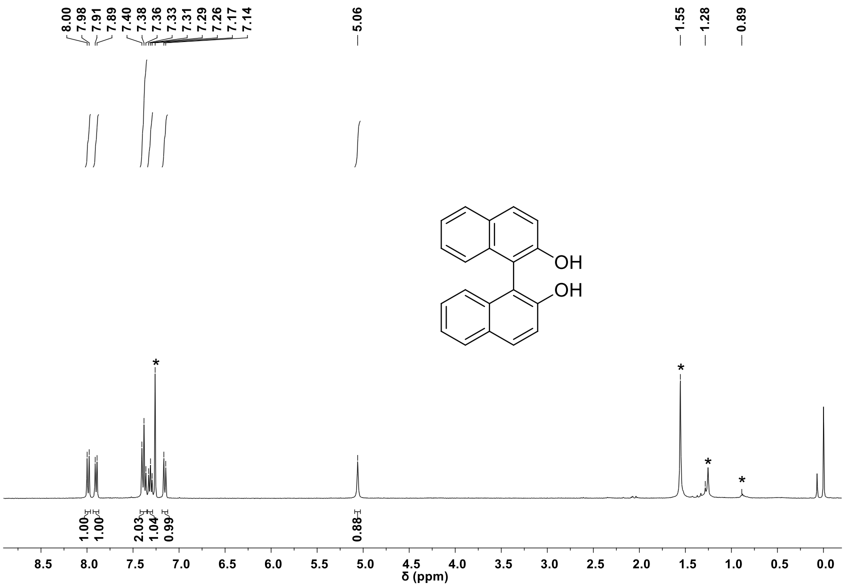

Supplementary Figure 8. ^{1}H NMR spectra of the 1,1-bi-2-naphthol product isolated from the precipitant in $CDCl_3$.

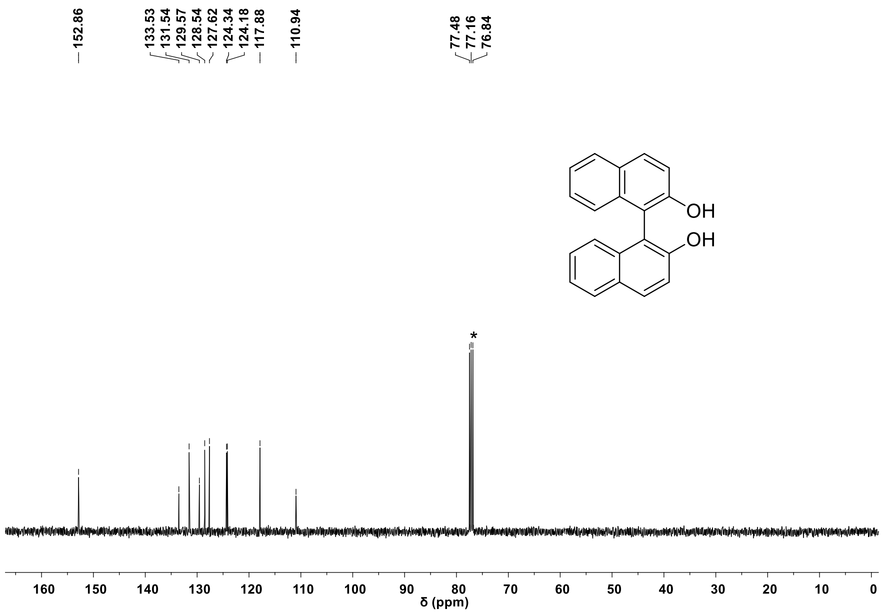

Supplementary Figure 9. ^{13}C NMR spectra of the 1,1-bi-2-naphthol product isolated from the precipitant in $CDCl_3$.

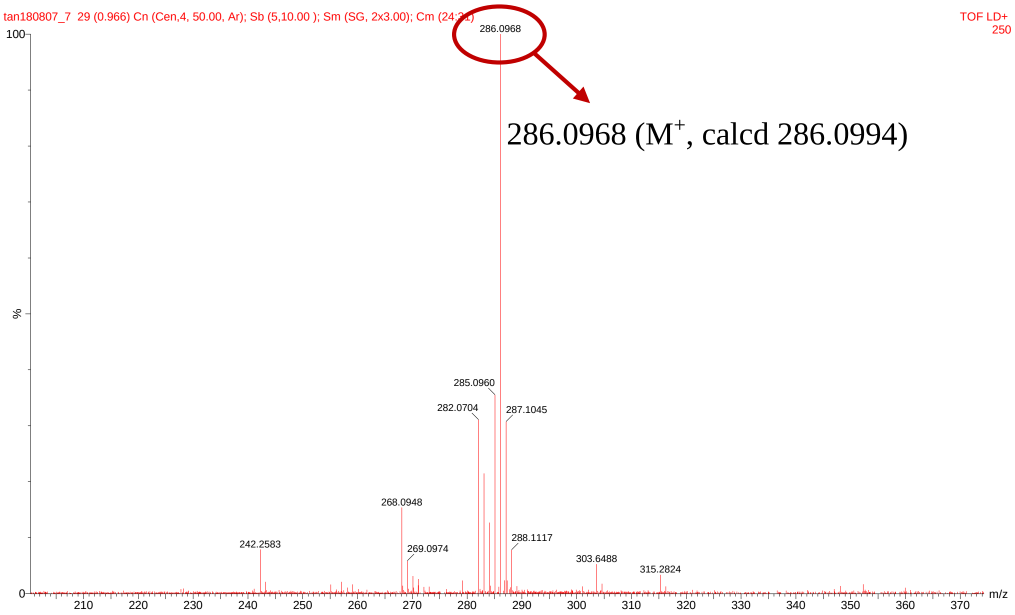

Supplementary Figure 10. HRMS (MALDI-TOF) spectrum of the 1,1-bi-2-naphthol product isolated from the precipitant.

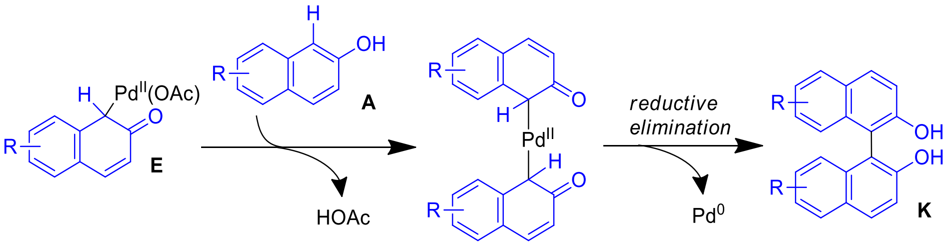

Supplementary Figure 11. A possible pathway for the formation of 1,1-bi-2-naphthol.[4]

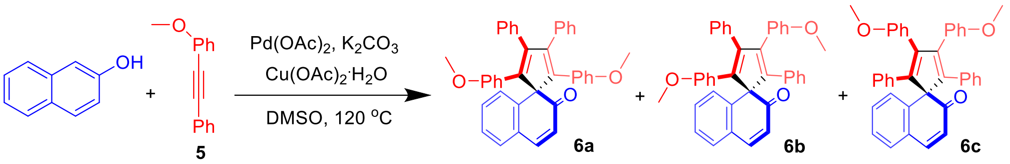

Supplementary Figure 12. Synthetic route to model compound **6**.

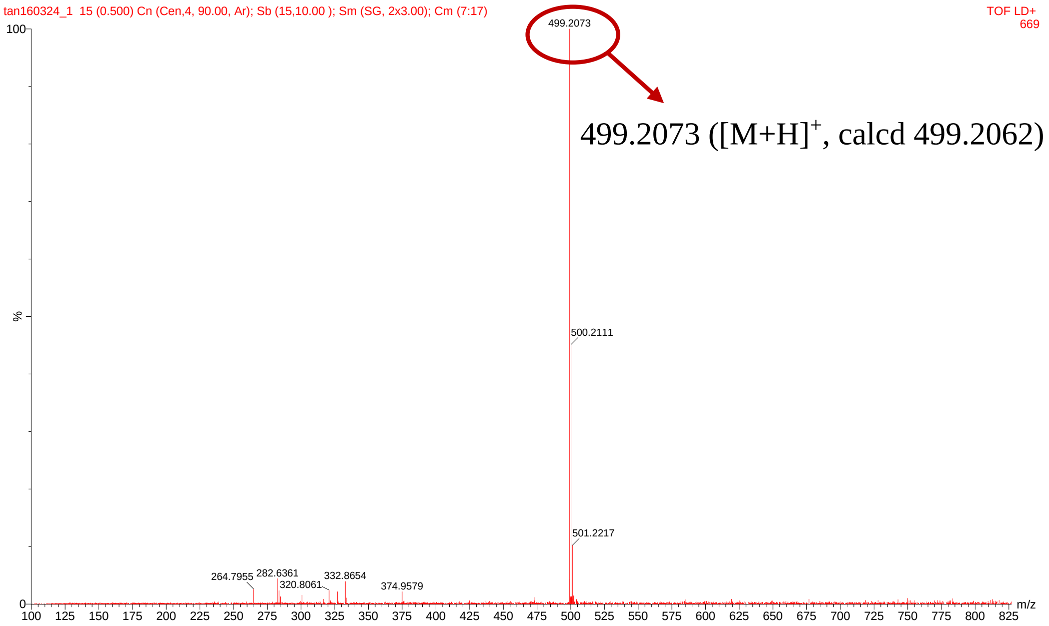

Supplementary Figure 13. HRMS (MALDI-TOF) spectrum of **4**.

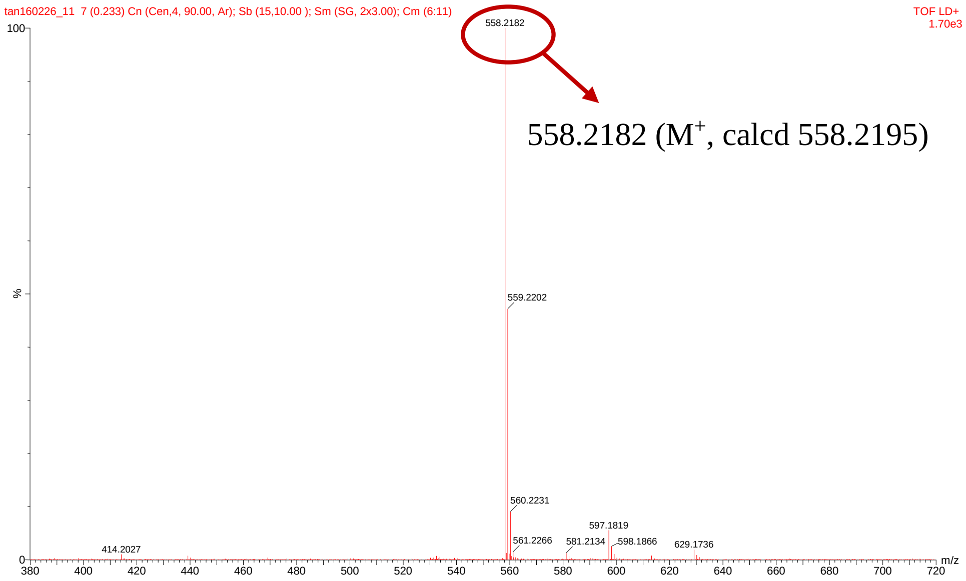

Supplementary Figure 14. HRMS (MALDI-TOF) spectrum of **6**.

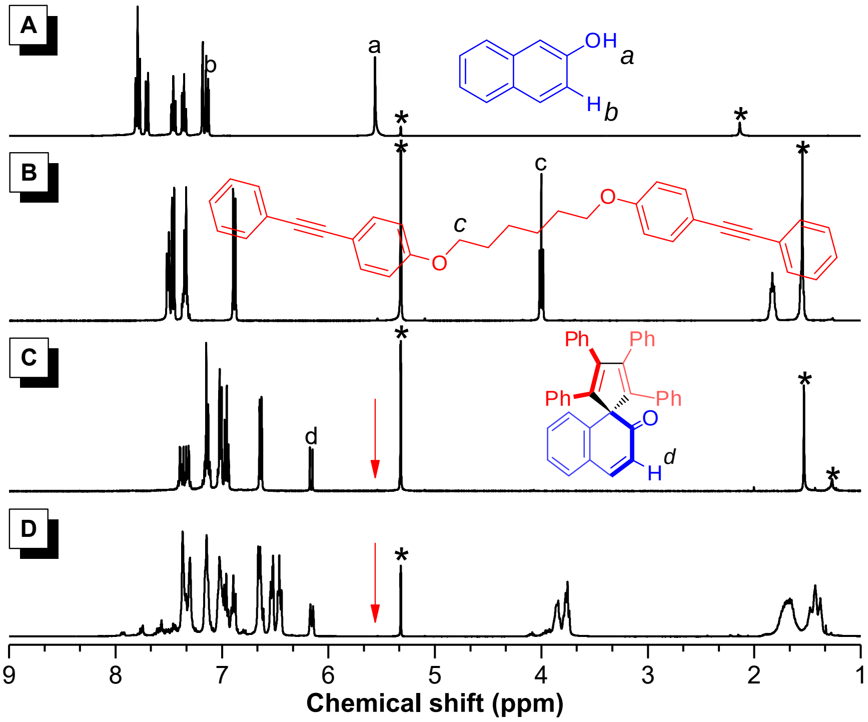

Supplementary Figure 15. ^{1}H NMR spectra of (A) **1a**, (B) **2a**, (C) **4** and (D) P**1a**/**2a** (sample taken from Table 1, entry 5) in CD_2Cl_2.

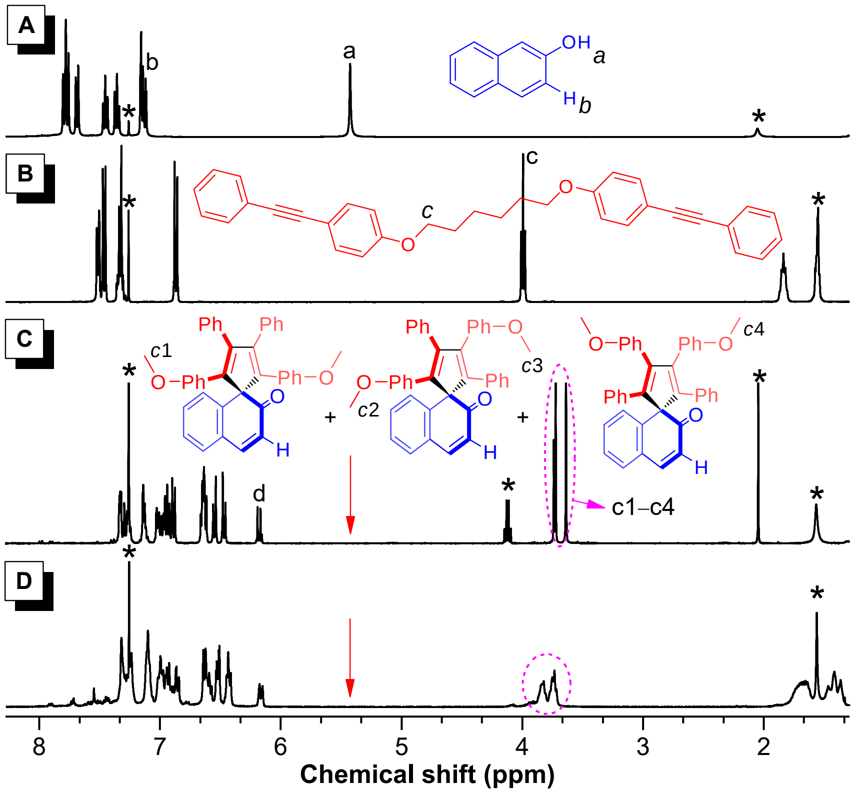

Supplementary Figure 16. ^{1}H NMR spectra of (A) **1a**, (B) **2a**, (C) **6** and (D) P**1a**/**2a** (sample taken from Table 1, entry 5) in $CDCl_3$.

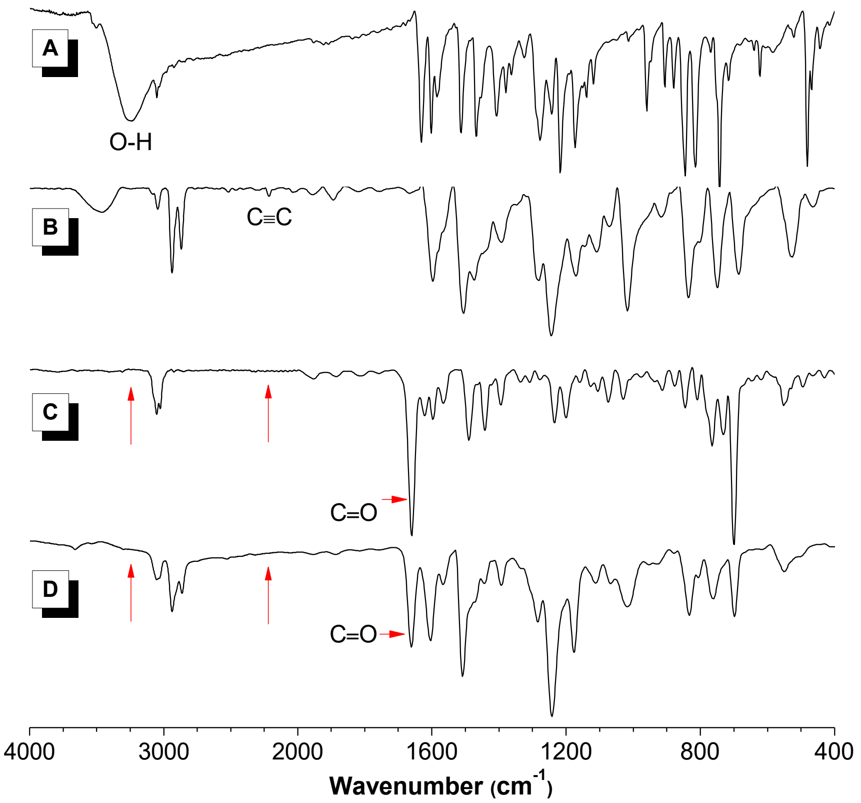

Supplementary Figure 17. IR spectra of (A) **1a**, (B) **2a**, (C) **4**, and (D) **P1a/2a** (sample taken from Table 1, entry 5).

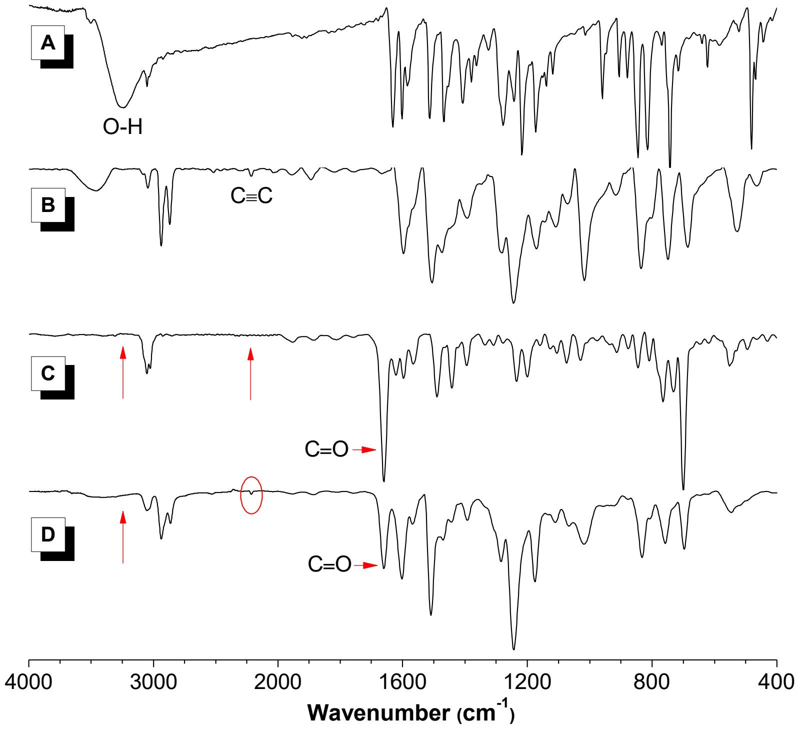

Supplementary Figure 18. IR spectra of (A) **1a**, (B) **2a**, (C) **4**, and (D) **P1a/2a** (sample taken from Table 1, entry 2).

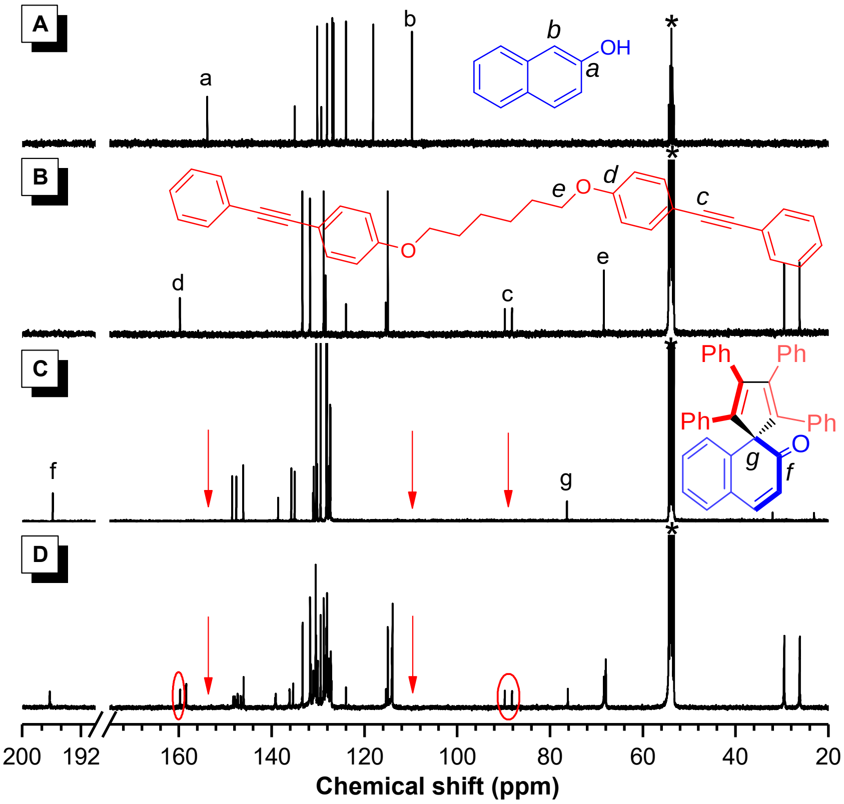

Supplementary Figure 19. ^{13}C NMR spectra of (A) **1a**, (B) **2a**, (C) **4** and (D) P**1a**/**2a** (sample taken from Table 1, entry 2) in CD_2Cl_2.

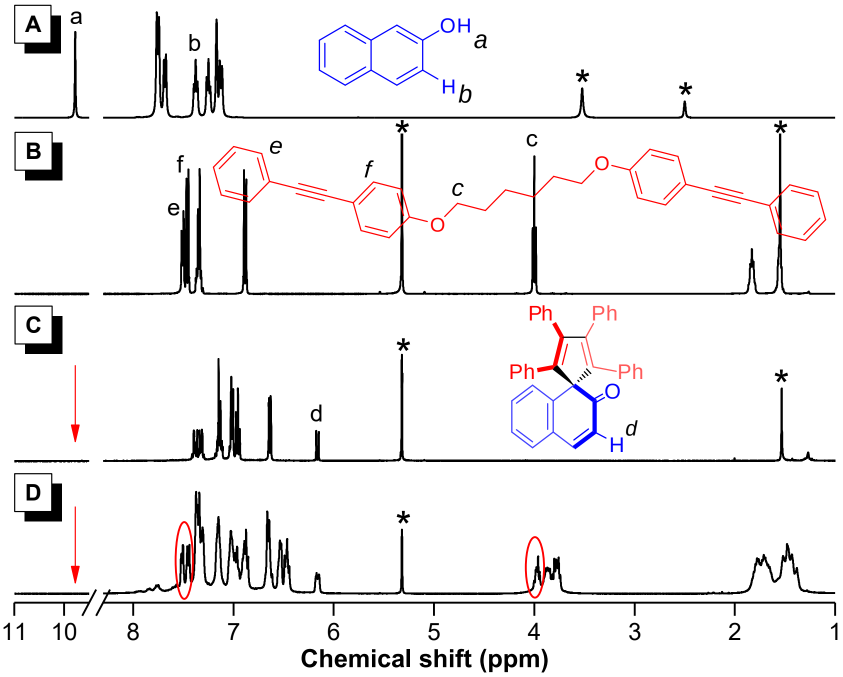

Supplementary Figure 20. ^{1}H NMR spectra of (A) **1a**, (B) **2a**, (C) **4** and (D) P**1a**/**2a** (sample taken from Table 1, entry 2) in CD_2Cl_2.

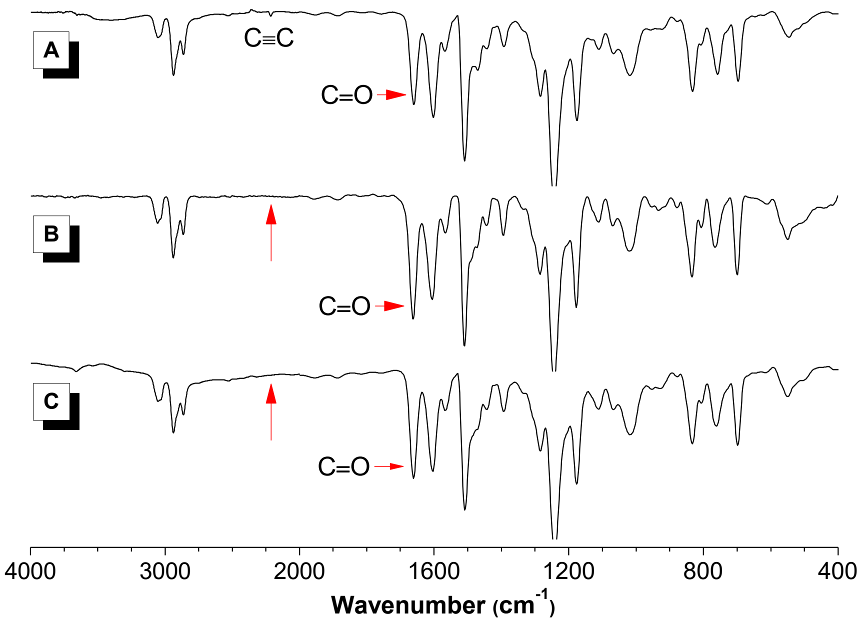

Supplementary Figure 21. IR spectra of (A) telechelic polymer P**1a**/**2a**, (B) extended P**1a**/**2a**, and (C) P**1a**/**2a** (sample taken from Table 1, entry 5).

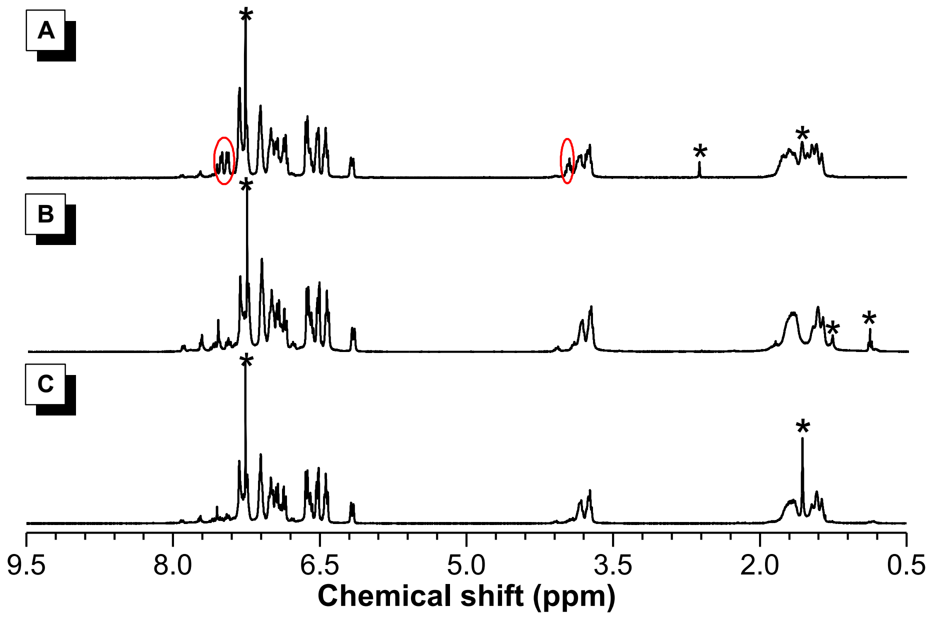

Supplementary Figure 22. ^{1}H NMR spectra of (A) telechelic polymer P**1a**/**2a**, (B) extended P**1a**/**2a**, and (C) P**1a**/**2a** (sample taken from Table 1, entry 5) in $CDCl_3$.

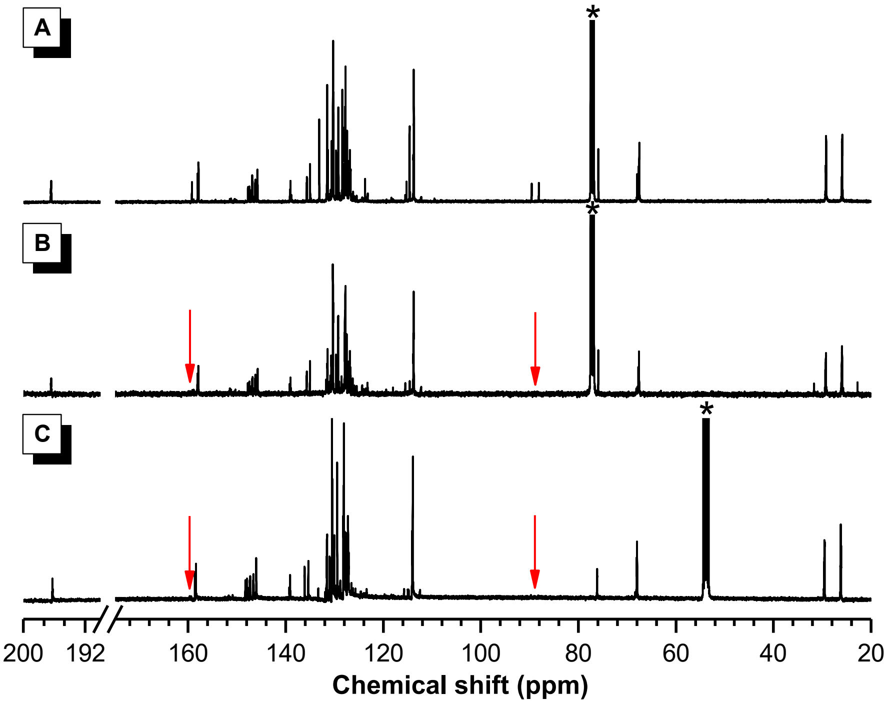

Supplementary Figure 23. ^{13}C NMR spectra of (A) telechelic polymer P**1a**/**2a**, (B) extended P**1a**/**2a**, and (C) P**1a**/**2a** (sample taken from Table 1, entry 5) in $CDCl_3$.

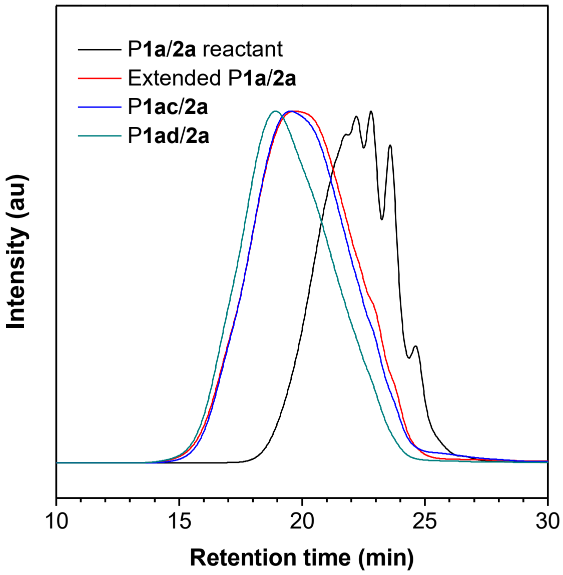

Supplementary Figure 24. GPC curves of telechelic polymer P**1a**/**2a** and its polymerization products.

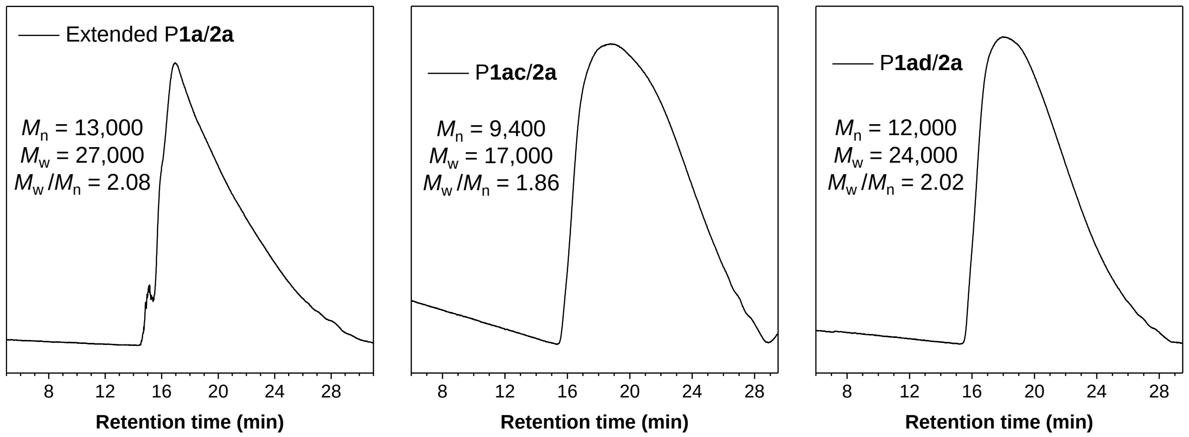

Supplementary Figure 25. GPC traces of the polymerization products of telechelic polymer P**1a**/**2a** (measured in DMF containing 0.1 M LiBr using a MALLS detector).

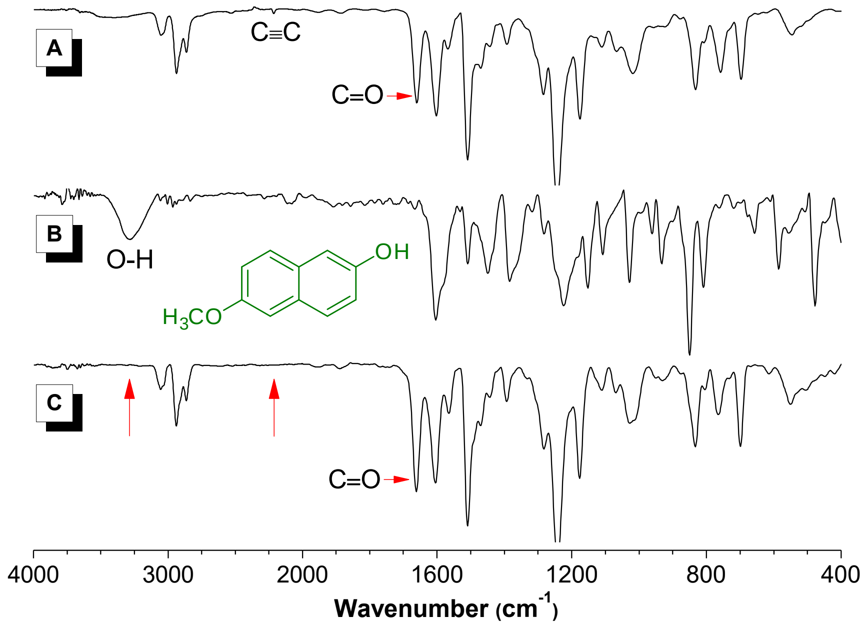

Supplementary Figure 26. IR spectra of (A) telechelic polymer P**1a**/**2a**, (B) **1c**, and (C) P**1ac**/**2a**.

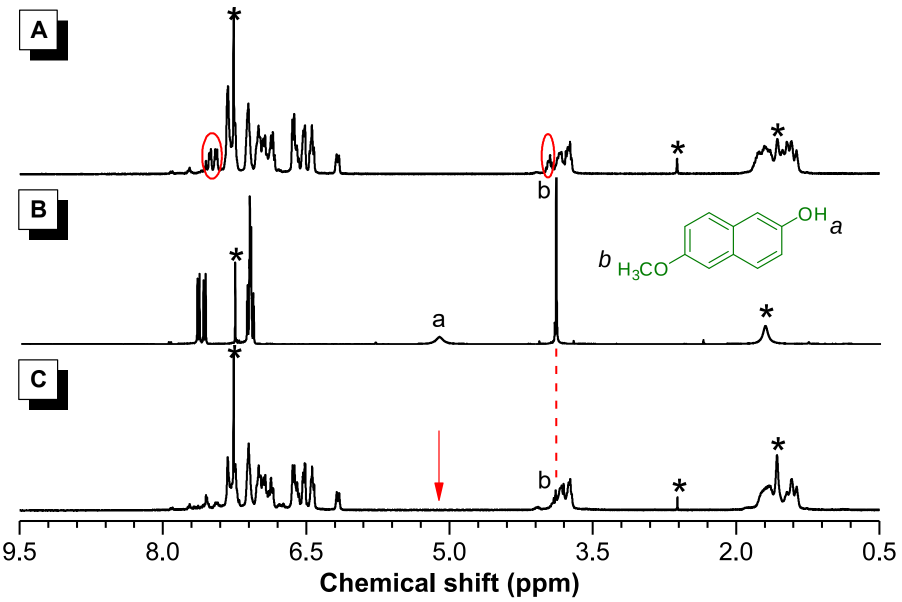

Supplementary Figure 27. ^{1}H NMR spectra of (A) telechelic polymer P**1a**/**2a**, (B) **1c**, and (C) P**1ac**/**2a** in $CDCl_3$.

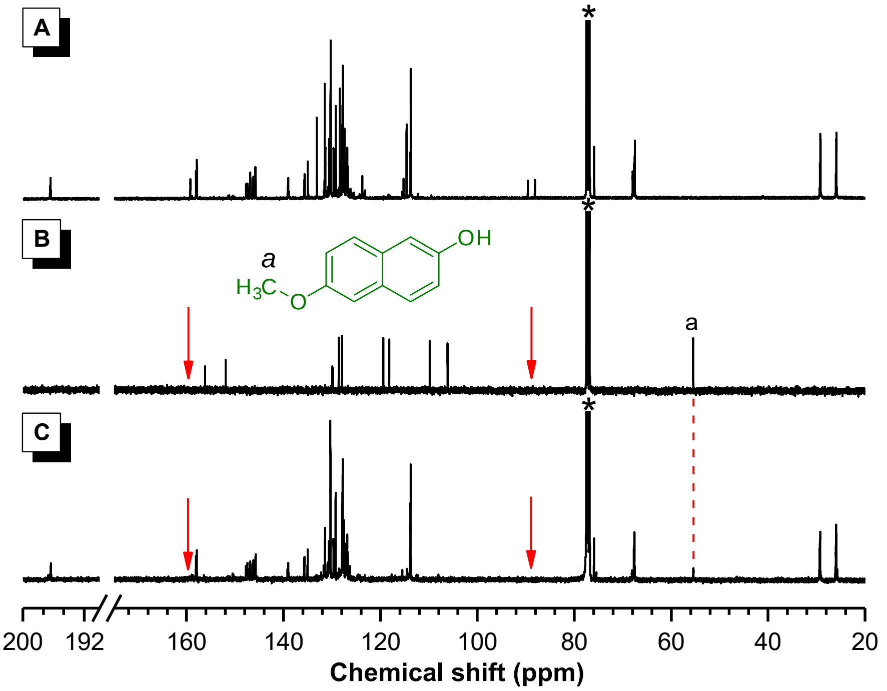

Supplementary Figure 28. ^{13}C NMR spectra of (A) telechelic polymer P**1a**/**2a**, (B) **1c**, and (C) P**1ac**/**2a** in $CDCl_3$.

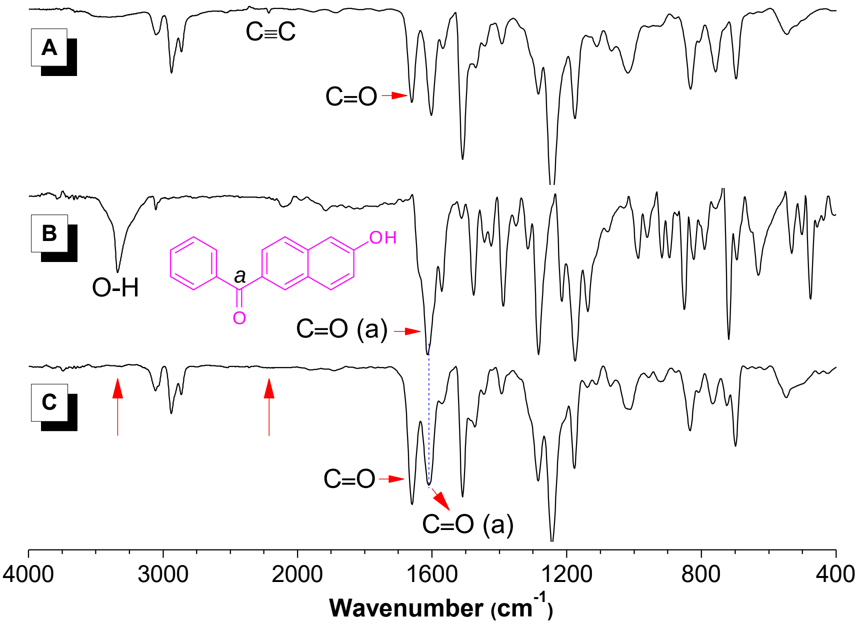

Supplementary Figure 29. IR spectra of (A) telechelic polymer P**1a**/**2a**, (B) **1d**, and (C) P**1ad**/**2a**.

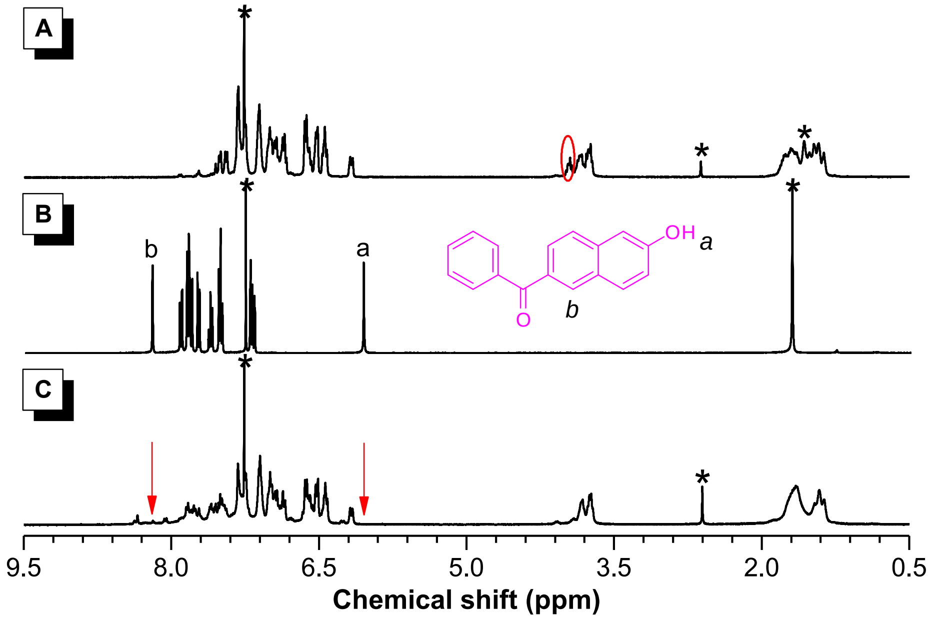

Supplementary Figure 30. ^{1}H NMR spectra of (A) telechelic polymer P**1a**/**2a**, (B) **1d**, and (C) P**1ad**/**2a** in $CDCl_3$.

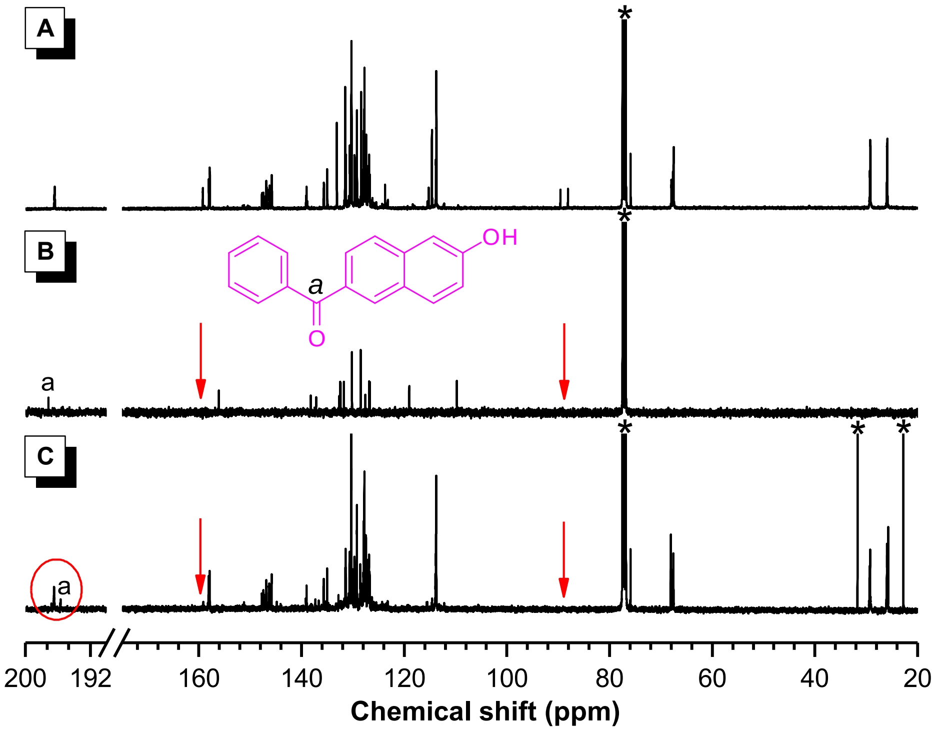

Supplementary Figure 31. ^{13}C NMR spectra of (A) telechelic polymer P**1a**/**2a**, (B) **1d**, and (C) P**1ad**/**2a** in $CDCl_3$.

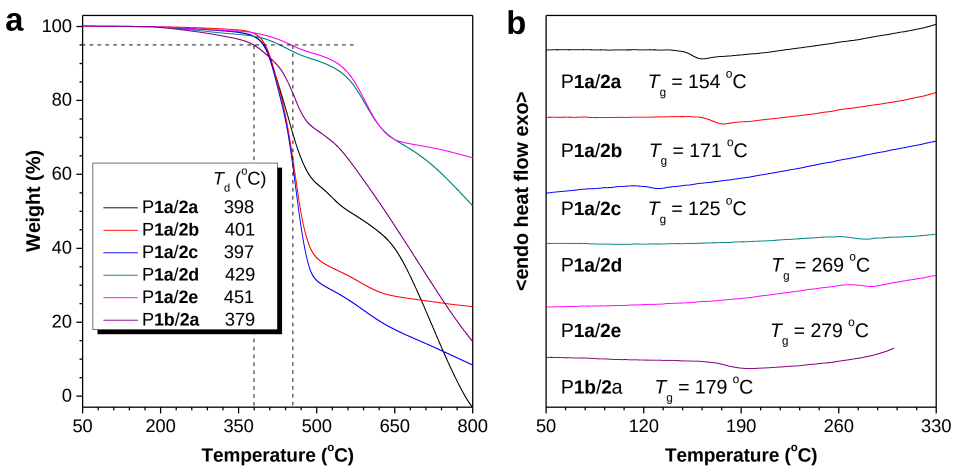

Supplementary Figure 32. (**a**) TGA curves and (**b**) DSC thermograms during the second heating cycle of P**1a**–**b**/**2a**–**e** recorded under nitrogen at a heating rate of 10 °C/min. T_d = decomposition temperature at 5% weight loss; T_g = glass transition temperature.

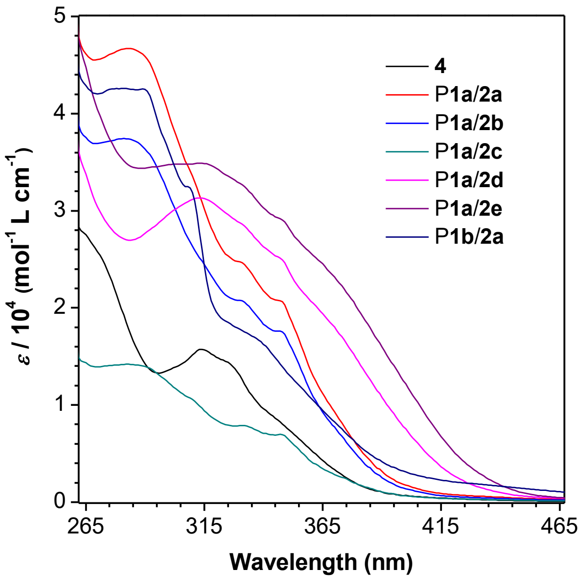

Supplementary Figure 33. Absorption spectra of **P1a–b/2a–e** in THF solutions. Solution concentration: 10 μM.

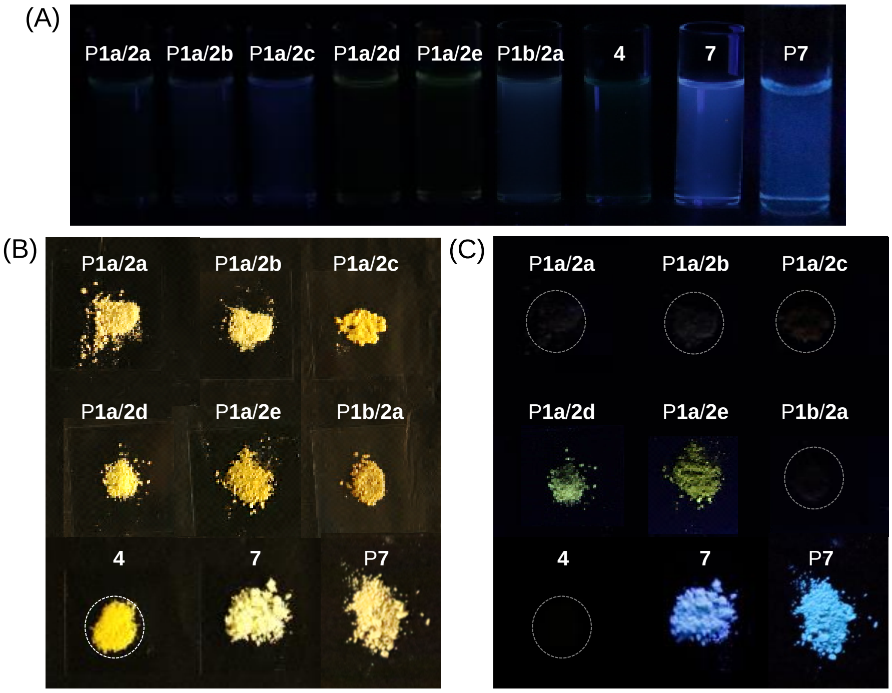

Supplementary Figure 34. The photos of the powder of polymers and model compounds taken under (A) room light and (B) 365-nm UV irradiation from a hand-held UV lamp.

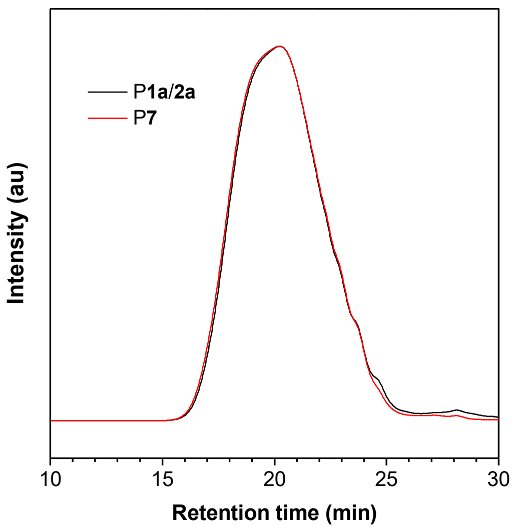

Supplementary Figure 35. GPC curves of P**1a**/**2a** and its reduced product P**7**.

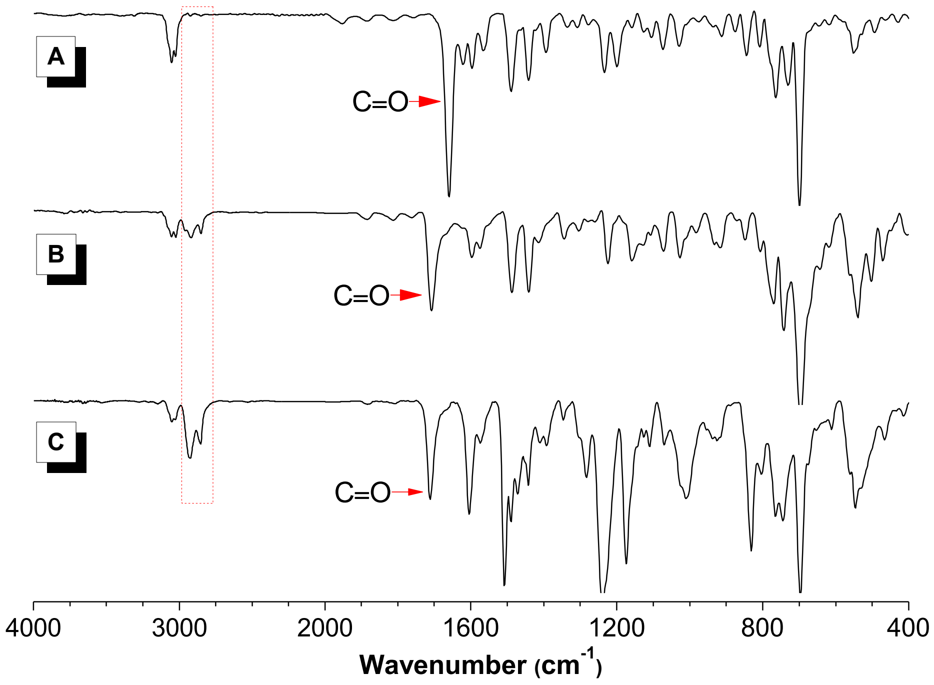

Supplementary Figure 36. IR spectra of (A) **4**, (B) **7**, and (C) P**7**.

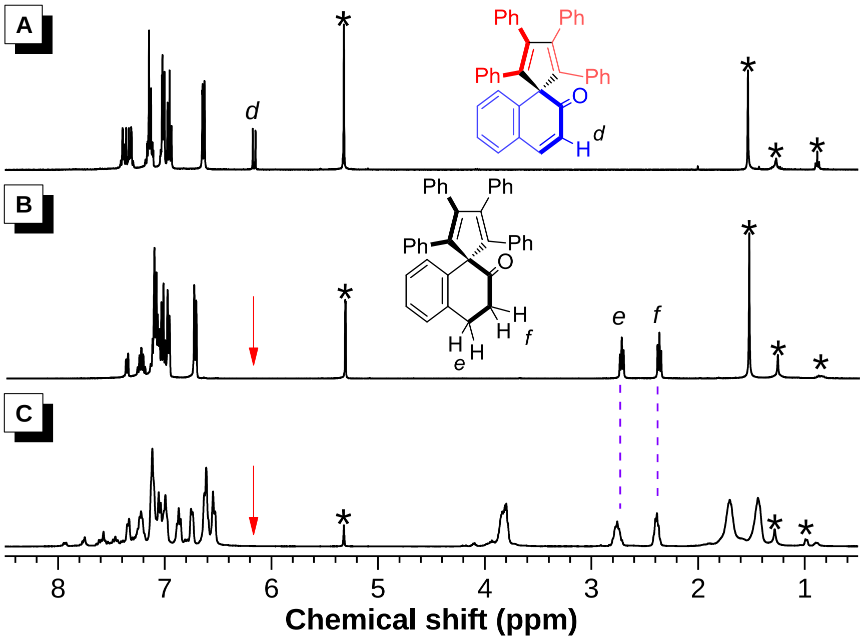

Supplementary Figure 37. ^{1}H NMR spectrum of (A) **4**, (B) **7**, and (C) P**7** in CD_2Cl_2.

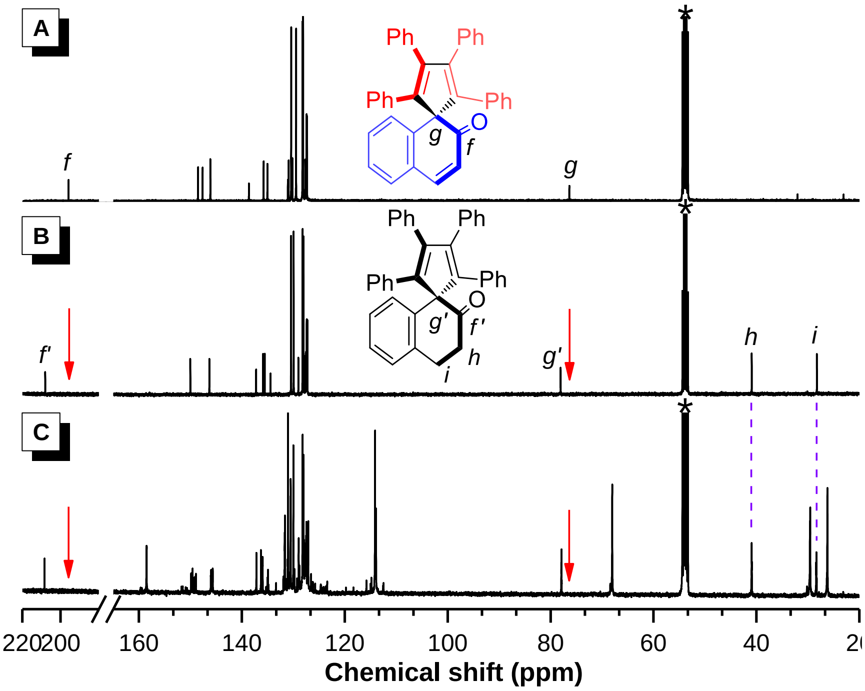

Supplementary Figure 38. ^{13}C NMR spectrum of (A) **4**, (B) **7**, and (C) P**7** in CD_2Cl_2.

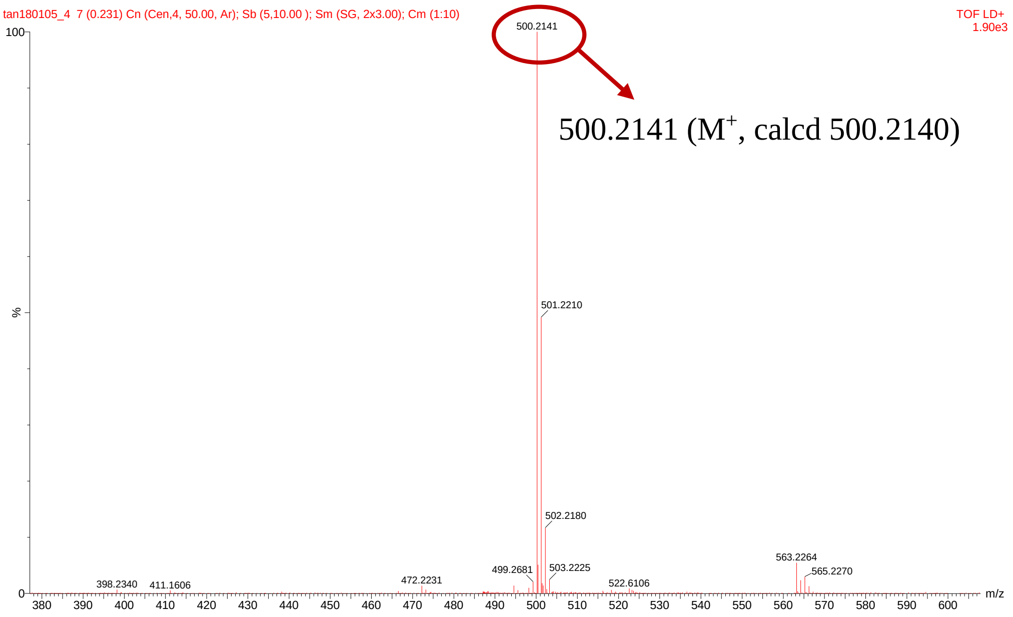

Supplementary Figure 39. HRMS (MALDI-TOF) spectrum of **7**.

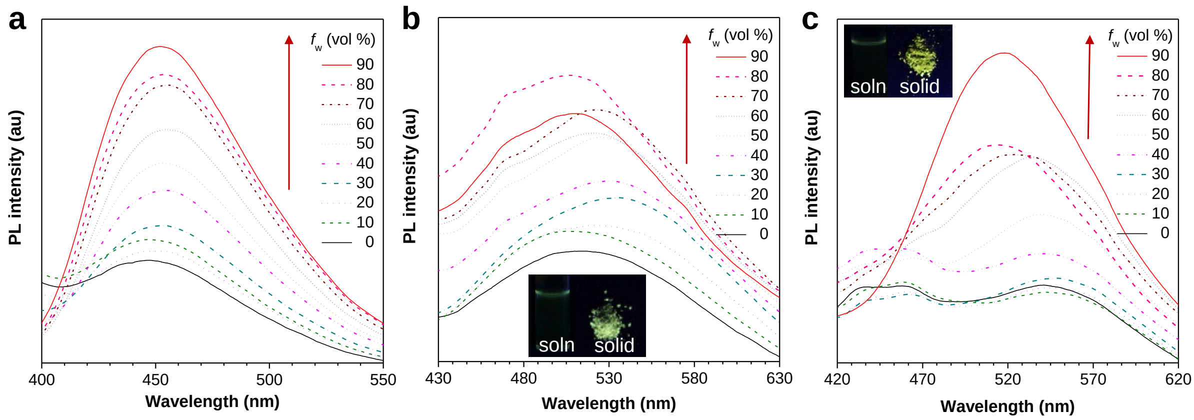

Supplementary Figure 40. PL spectra of (**a**) P**7**, (**b**) P**1a**/**2d** and (**c**) P**1a**/**2e** in THF and THF/water mixtures with different water fractions (f_W). Solution concentration: 10 μM; excitation wavelength: 320 nm for P**7** and 350 nm for P**1a**/**2d** and P**1a**/**2e**. Inset: fluorescent images of the THF solution and powder of P**1a**/**2d** and P**1a**/**2e** taken under a hand-held UV lamp.

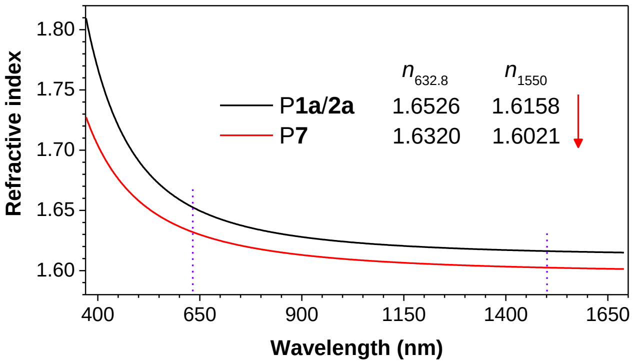

Supplementary Figure 41. Wavelength dependence of refractive indices of thin films of P**1a**/**2a** and P**7**.

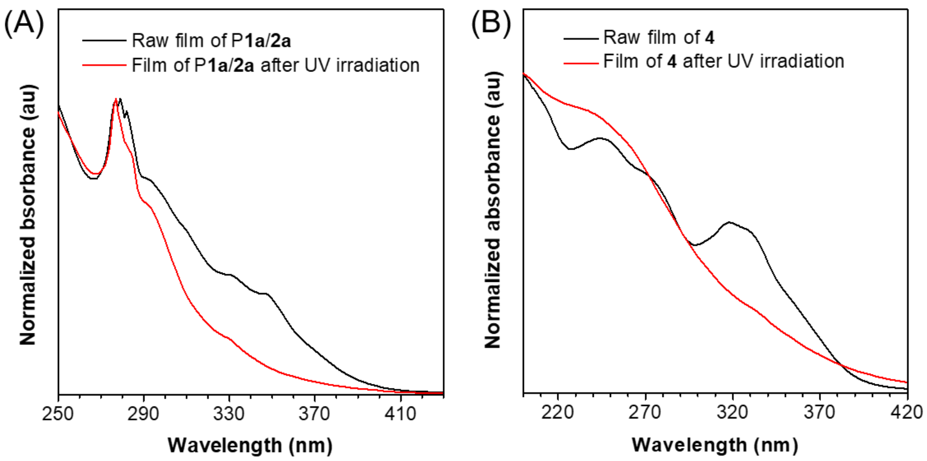

Supplementary Figure 42. UV-vis absorption spectra of the drop-casting thin film of (A) P**1a**/**2a** and (B) model compound **4** before and after UV irradiation.

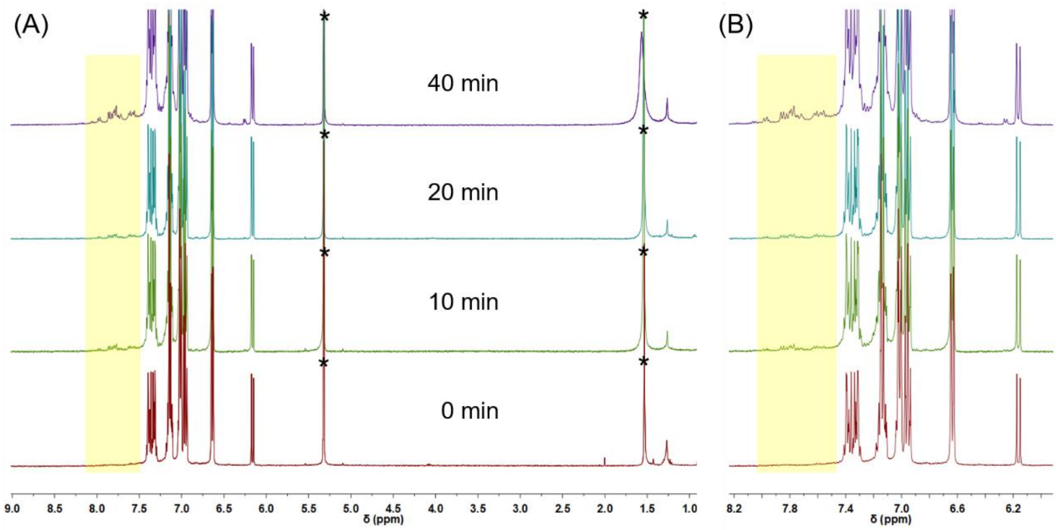

Supplementary Figure 43. (A) ^{1}H NMR spectra of model compound **4** under different UV irradiation time and (B) their enlarged spectra. The ^{1}H NMR spectra were measured in CD_2Cl_2.

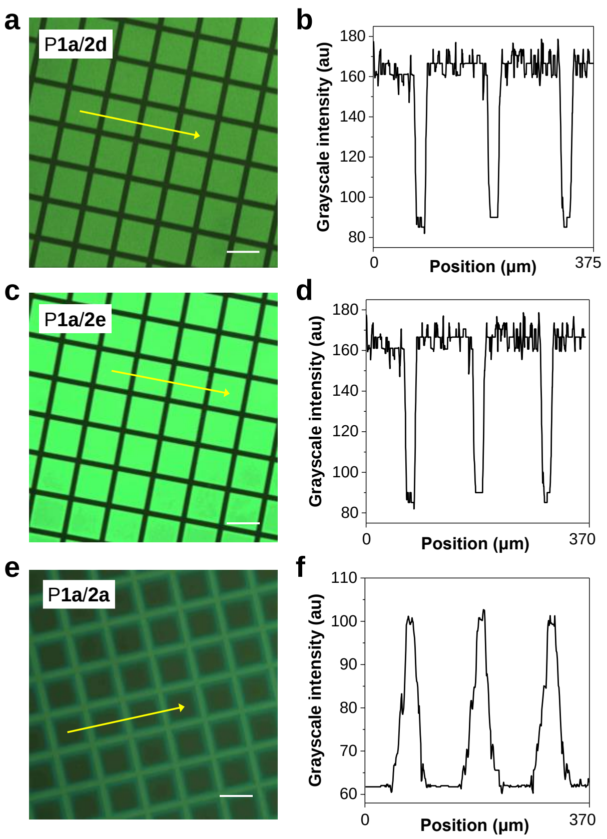

Supplementary Figure 44. Photographs of the fluorescent photopatterns of (**a**) P**1a**/**2d**, (**c**) P**1a**/**2e**, and (**e**) P**1a**/**2a** taken under 330–380 nm UV illumination using a fluorescent microscope. (**b**, **d**, and **f**) The associated grayscale intensity profile of the arrowed area in the fluorescent images. Scale bar = 100 μm.

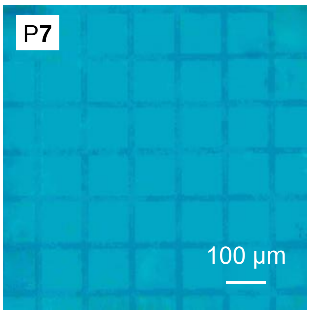

Supplementary Figure 45. The photograph of the photopattern of P**7** taken under normal room light using an optical microscope.

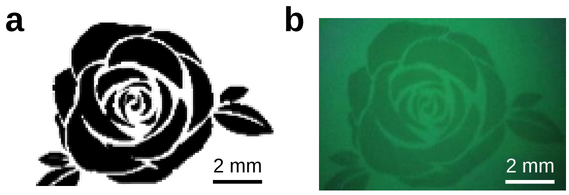

Supplementary Figure 46. (**a**) The flower-like photomask used for the photopatterning process. (**b**) Fluorescent photopatterns of P**1a**/**2a** taken under UV irradiation at 365 nm using a camera.

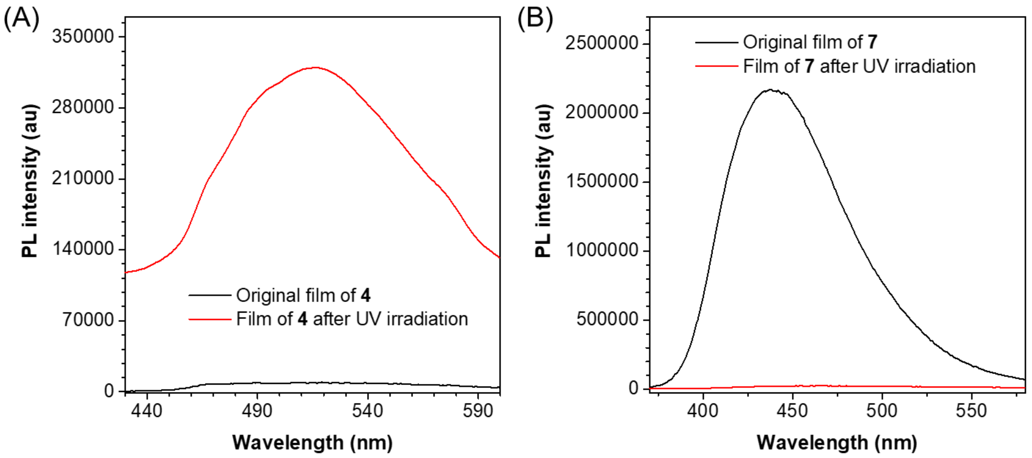

Supplementary Figure 47. PL spectra of the drop-casting thin film of (A) model compound **4** and (B) model compound **7** before and after UV irradiation.

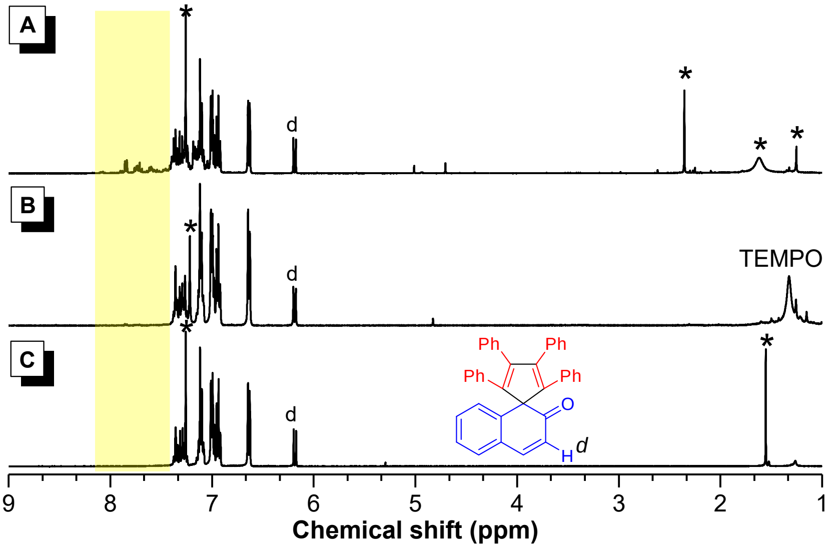

Supplementary Figure 48. ^1H NMR spectra of the model compound **4** (A) after irradiating its toluene solution by UV light for 3 h, (B) after irradiating its TEMPO-containing toluene solution by UV light for 3 h, and (C) without UV irradiation. The ^1H NMR spectra were measured in $CDCl_3$.

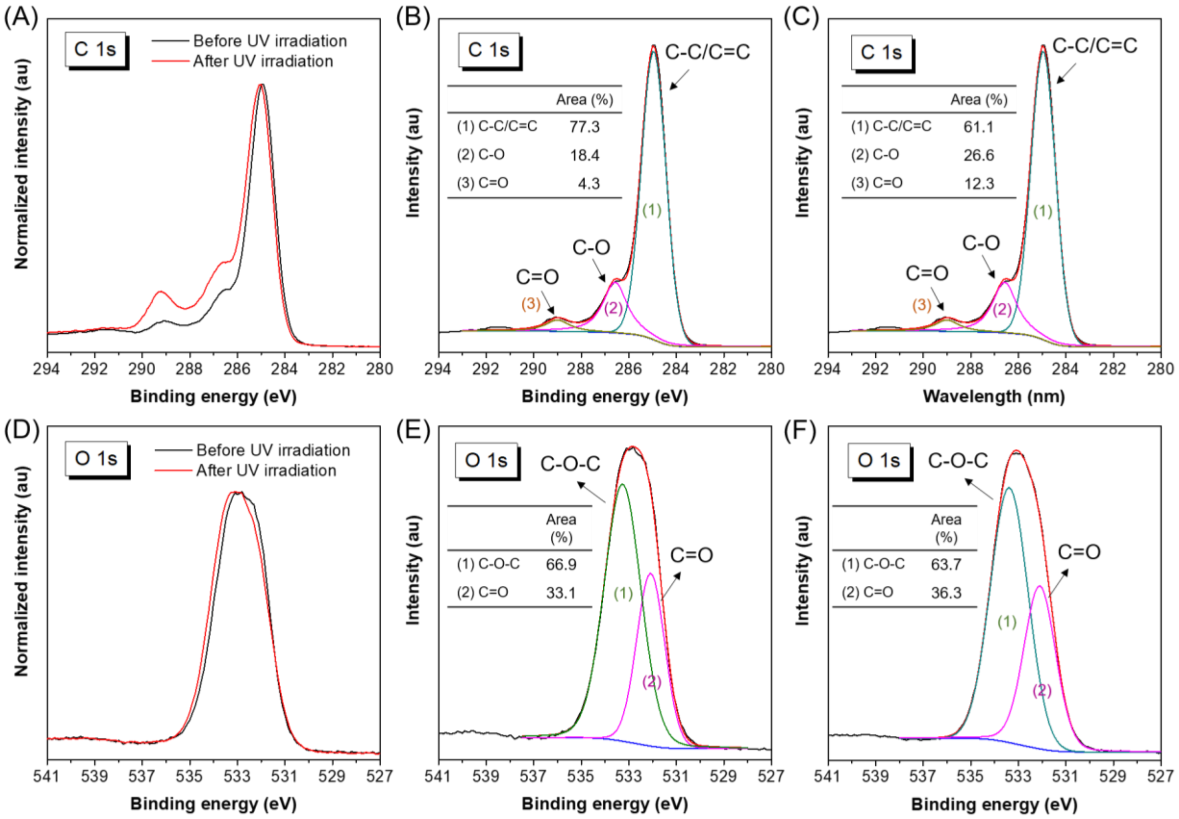

Supplementary Figure 49. High-resolution XPS spectra of C 1s and O 1s of P**1a**/**2a** film before and after UV irradiation. (B and E) XPS spectra before UV irradiation. (C and F) XPS spectra after 40-min UV irradiation.

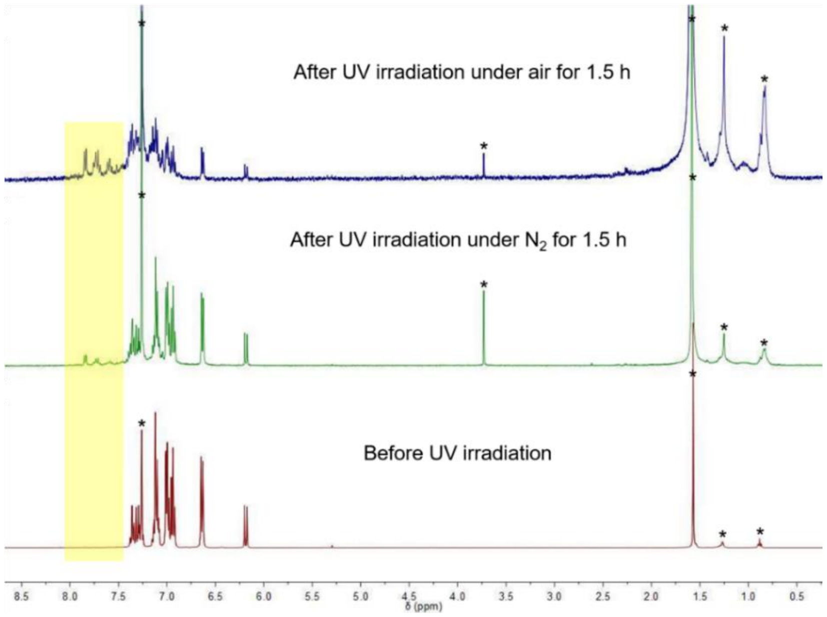

Supplementary Figure 50. ^{1}H NMR spectra of model compound **4** before UV irradiation and after UV irradiation under air and under N_2, respectively. The ^{1}H NMR spectra were measured in $CDCl_3$.

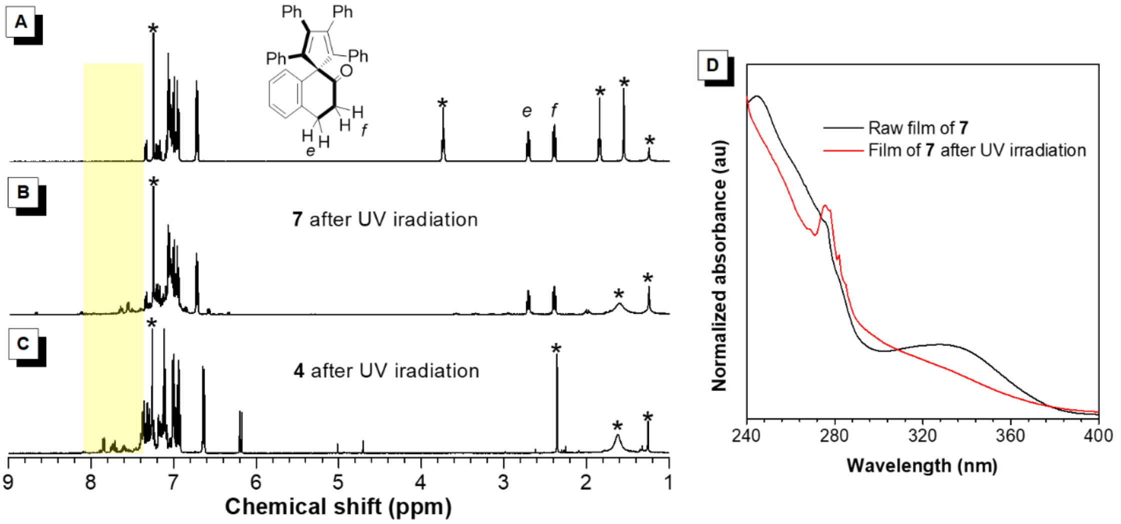

Supplementary Figure 51. (A and B) ^{1}H NMR spectra of **7** before and after UV irradiation. Detailed procedures: the film of 7 was exposed to UV light for 1.5 h and then washed by $CDCl_3$ to measure the ^{1}H NMR spectrum. (C) ^{1}H NMR spectrum of **4** after UV irradiation. All the ^{1}H NMR spectra were measured in $CDCl_3$. (D) Absorption spectra of model compound **7** before and after UV irradiation for 1.5 h.

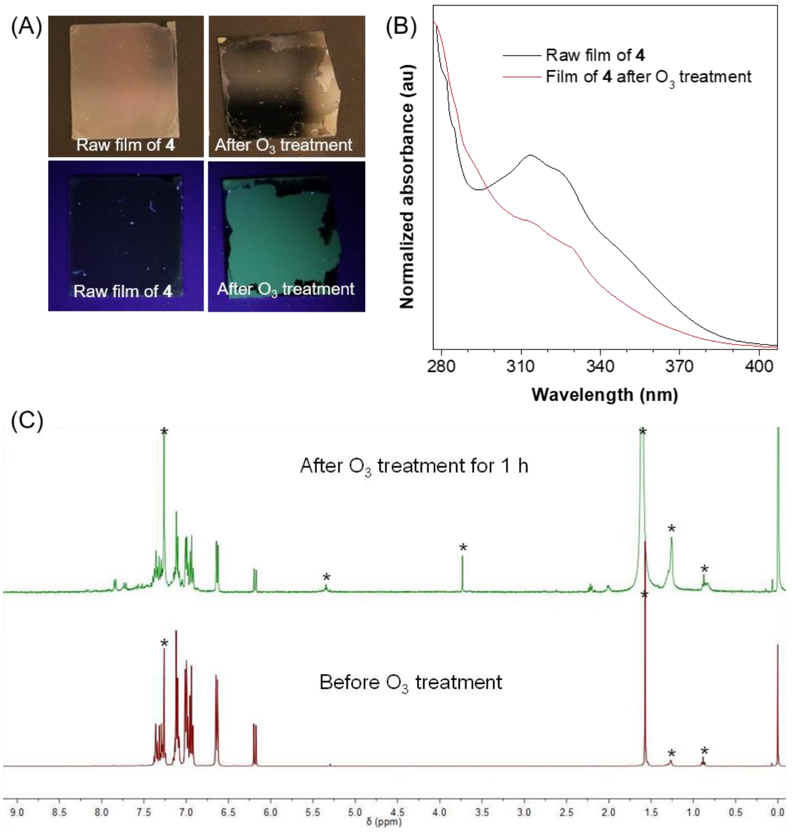

Supplementary Figure 52. (A) Photos of the thin films of model compound **4** before and after O_3 treatment taken under normal room light (upper panel) and 365 nm UV irradiation (lower panel), respectively. (B) UV spectra of model compound **4** before and after O_3 treatment. (C) ^{1}H NMR spectra of model compound **4** before and after O_3 treatment for 1h. The ^{1}H NMR spectra were measured in $CDCl_3$.

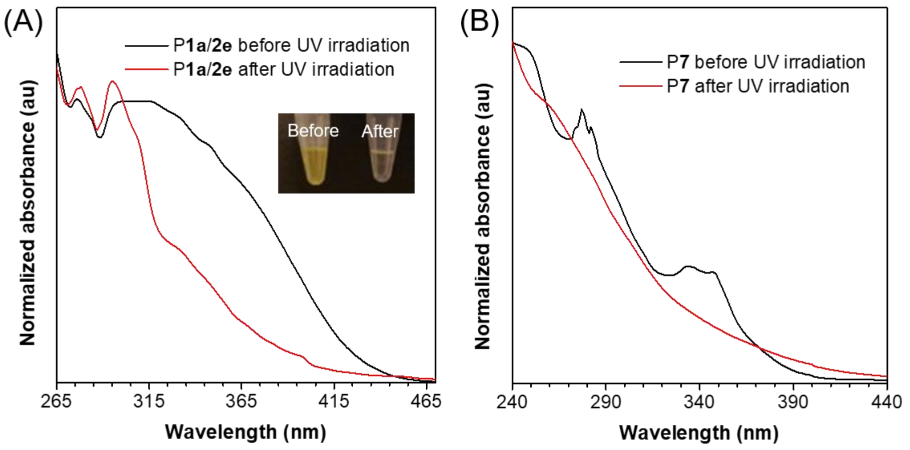

Supplementary Figure 53. UV-vis absorption spectra of the thin films of (A) P**1a**/**2e** and (B) P**7** before and after UV irradiation for 1.5 h.

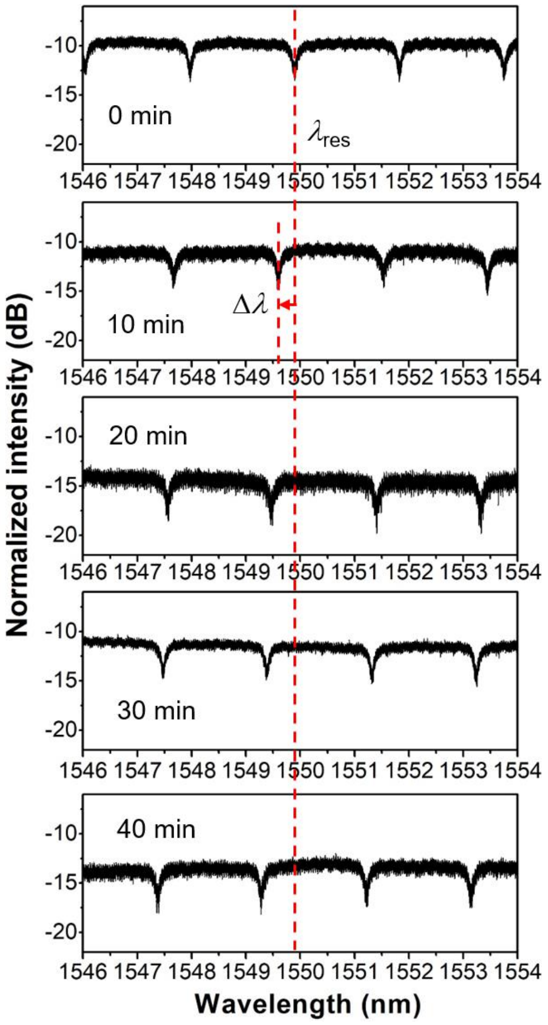

Supplementary Figure 54. Measured transmission spectra of microring D4 near 1550 nm wavelength in TE polarization with UV exposure duration varying from 0 to 40 minutes at an interval of 10 minutes.

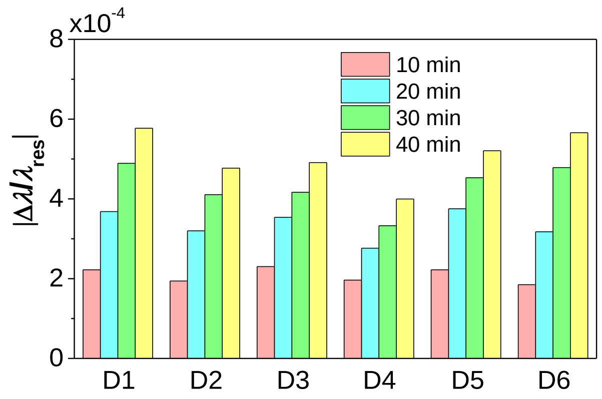

Supplementary Figure 55. Extracted $|\Delta\lambda/\lambda_{res}|$ upon different UV exposure durations of all the six devices in TE polarization.

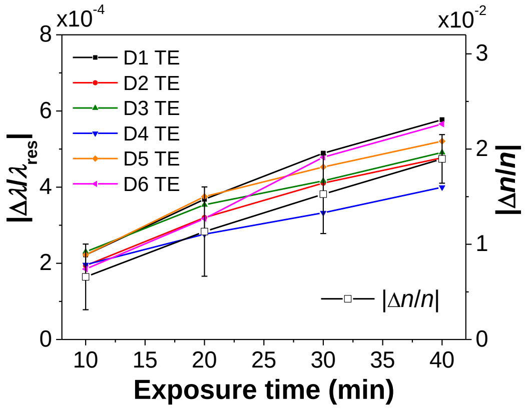

Supplementary Figure 56. Comparison between the extracted $|\Delta\lambda/\lambda_{res}|$ of the microrings in TE polarization and the extracted $|\Delta n/n|$ of the polymer films as a function of the UV exposure duration. The error bars indicate the standard deviations.

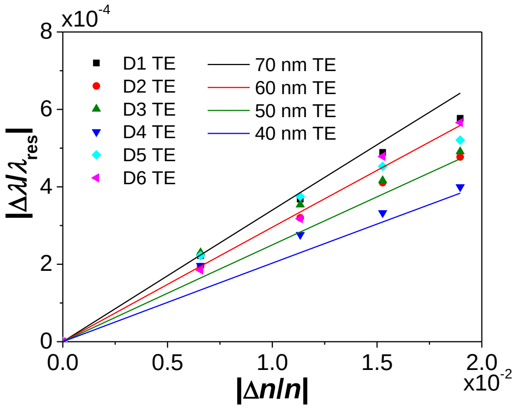

Supplementary Figure 57. Comparison between the measured $|\Delta\lambda/\lambda_{res}|$ (symbols) of the six devices and the calculated $|\Delta\lambda/\lambda_{res}|$ (lines) in TE polarization with a polymer thickness ranging from 40 nm to 70 nm as a function of the measured $|\Delta n/n|$ of the polymer films upon different UV exposure durations.

Supplementary Discussion

Mechanistic study on the UV-responsive refractive index. It is known that refractive index (RI) can be influenced by the polarizability of the components composing the polymer repeating unit and the presence of highly polarizable π-conjugated functionalities are beneficial for increasing the refractive indices of polymers.[5] Therefore, the decrease in electronic conjugation could result in the decrease in RI. For example, the RI of P**7** is obviously lower than that of P**1a**/**2a** (Supplementary Table 6 and Supplementary Figure 41), which indicated that the conversion of the unsaturated C=C to saturated C–C can lead to a decrease in RI due to the decreased electronic conjugation.

There are multiple reactive sites in the multisubstituted spirocyclic polymer structures that could be photo-oxidized under strong UV irradiation, which will reduce the electronic conjugation and thus lead to the decrease in RI. The absorption spectrum of P**1a**/**2a** were then measured before and after UV irradiation. Supplementary Figure 42A suggested that the strong UV irradiation could indeed lead to a decrease in the electronic conjugation of P**1a**/**2a**. Due to the intrinsic complexity and polydispersity of polymers, the UV responsiveness of model compound **4** was also investigated to assist the mechanistic understanding. The absorption spectra of its thin films onto quartz plates were measured before and after UV irradiation. As shown in Supplementary Figure 42B, the absorption spectrum significantly changed after UV irradiation. The absorption peak at 318 nm disappeared after UV irradiation, which is also indicative of the decreased electronic conjugation. Therefore, the photo-induced RI decrease of P**1a**/**2a** is possibly due to the decrease in its electronic conjugation under the irradiation of strong UV light.

To gain insight into the structural change, the films of **4** were exposed to UV light for different time and then washed by CD_2Cl_2 to do the ^{1}H NMR characterization. The obtained ^{1}H NMR spectra shown in Supplementary Figure 43 implied that the intense UV irradiation indeed changed the chemical structure of **4** because some new peaks were observed in the aromatic proton region of the ^{1}H NMR spectra of the irradiated samples. Furthermore, these new resonance peaks became more and more obvious with the increase in UV irradiation time. We have tried very hard to analyze the photoreaction products, but the photoreaction of **4** seems to be very complicated under such a strong UV light source and the exact structures of the photoreaction products are still difficult to be determined currently.

Mechanistic study on the UV-responsive fluorescence. A radical marker 2,2,6,6-tetramethylpiperidin-1-yl)oxyl (TEMPO) was employed to test whether the photochemical reaction of the present system involved free radical intermediates. The toluene solution of **4** (5 mg/3 mL) and the toluene solution (3 mL) of model compound **4** (5 mg) together with 20 equivalents of TEMPO (31.3 mg) in quartz cells were simultaneously irradiated by UV light for 3 h. Afterward, the toluene solvent was removed and the solid was dissolved to measure the ^{1}H NMR. As summarized in Supplementary Figure 48, the ^{1}H NMR spectrum of the sample with the addition of TEMPO did not show the aforementioned new peaks in the aromatic proton region after UV irradiation, indicating that the photochemical reaction of this carbonyl-containing spirocyclic system may involve free radical intermediates. The generated free radicals can be immediately trapped by TEMPO and thus prevent the occurrence of further

reaction.

The high-resolution X-ray photoelectron spectroscopy (XPS) results of P**1a**/**2a** film clearly revealed the change in the surface of the sample after UV irradiation (Supplementary Figure 49). The C 1s spectra showed that the C-O and C=O band become higher after UV irradiation and meanwhile the proportion of the C-C/C=C band decreased. Therefore, photooxidation reactions possibly occurred after irradiating the P**1a**/**2a** film under strong UV light. The relative content of C=O and C-O signal in the C 1s and O 1s spectra indicated that the photooxidation process may generate both the C=O and C-O groups in the product structures.

The effect of oxygen on the photochemical reactions was then investigated by conducting the photoirradiation process of the film of **4** under air and under N_2, respectively. The ^{1}H NMR spectra shown in Supplementary Figure 50 suggested that the presence of oxygen is favorable for the proceeding of photoreactions. The resonance signal of the carbonyl-activated alkene proton of **4** (at ~δ 6.16) was obviously weakened after UV irradiation under air, and meanwhile some new resonance peaks were detected in the aromatic proton region. Therefore, the polymers are possible to undergo photooxidation reactions with the participation of the carbonyl-activated C=C double bonds under the intense UV irradiation.

Supplementary Figure 51 suggested that model compound **7** (without the carbonyl-activated C=C double bond) can also undergo photochemical reactions under intense UV irradiation. Some new resonance peaks were detected in the aromatic proton region of the ^{1}H NMR spectrum of the UV-irradiated **7** although the new signals seem to be less than those of **4**. These results suggested that besides the carbonyl-activated C=C double bond, the other reactive sites of this unique multisubstituted spirocyclic structure may also be reacted under the irradiation of strong UV light.

To further testify the possible photooxidation mechanism, we then tried to oxidize the film of **4** using ozone as the oxidant and investigate the change in its photophysical properties. The thin film was fabricated by spin-coating the 1,2-dichloroethane solution of **4** onto the silicon wafers. As shown in Supplementary Figure 52A, the non-emissive film of **4** became fluorescent after the film was exposed to ozone (generated from an ozonator) for about 1 h. The changes in the UV and ^{1}H NMR spectrum of **4** after O_3 treatment (Supplementary Figure 52B and C) were similar to the results obtained after UV irradiation (Supplementary Figures 42, 43 and 50). Therefore, the UV-activated fluorescence of P**1a**/**2a** and P**1b**/**2a** is very likely due to the generation of luminescent species from their photooxidation reactions.

The absorption spectrum of P**1a**/**2e** and P**7** before and after UV irradiation suggested that UV irradiation could lead to a decrease in the electronic conjugation of the tetraphenylethylene (TPE)-containing polymers (P**1a**/**2e**) and the reduced polymer (P**7**) (see Supplementary Figure 53). There are many reactive sites in these polymer structures that could be photo-oxidized to result in the decreased electronic conjugation. Therefore, these polymers are possible to undergo photooxidation reactions under the irradiation of strong UV light.

The opposite photoresponsive behavior between P**1a**/**2d**–**e** and P**1a**/**2a** suggested that the TPE moiety may play a crucial role in the UV-quenched fluorescence of P**1a**/**2d**–**e**. It has been reported that the C=C group of TPE is photosensitive and can be photo-oxidized under intense UV irradiation.[6] Various TPE-containing polymers have been reported to show photo-oxidative bleaching.[7,8] We also tried to oxidize the TPE film using ozone (generated from an ozonator)

as the oxidant to study the oxidation-induced structure change in their luminescent properties. After exposure to ozone for about 1 h, the strong fluorescence of TPE film was found to be almost completely quenched. By contrast, the tetraphenyl-substituted spirocyclic compound (**4**) was found to show a phenomenon of UV-activated fluorescence (Supplementary Figure 47A) or oxidation-activated fluorescence (Supplementary Figure 52A). These results indicated that the photooxidation of the TPE moiety could play a leading role in the photobleaching behavior of the TPE-containing spiropolymers (P**1a**/**2d** and P**1a**/**2e**).

The investigation on the photoresponsiveness of model compound **7** (Supplementary Figures 47B and 51) suggested that the photobleaching behavior of P**7** is also related to the occurrence of photochemical reactions that can lead to a decrease in its electronic conjugation. Because 1,3-cyclopentadiene has been widely reported to be a photosensitive unit that can undergo photooxidation reactions to afford oxidized products with less conjugated structures,[9,10] it is reasonable to propose that the photobleaching behavior of P**7** may mainly result from the photooxidation of its AIE-active 1,2,3,4-tetraphenyl-1,3-cyclopentadiene (TPC) moiety.

Supplementary References

1. Deng, H. Q., et al. One-pot three-component tandem polymerization toward functional poly(arylene thiophenylene) with aggregation-enhanced emission characteristics. *Macromolecules* **47**, 4920–4929 (2014).
2. Zhang, Y., Zhao, E. G., Deng, H. Q., Lam, J. W. Y. & Tang, B. Z. Development of a transition metal-free polymerization route to functional conjugated polydiynes from a haloalkyne-based organic reaction. *Polym. Chem.* **7**, 2492–2500 (2016).
3. Zhou, X., et al. Piezofluorochromism and morphology of a new aggregation-induced emission compound derived from tetraphenylethylene and carbazole. *New J. Chem.* **36**, 685–693 (2012).
4. Gu, S. L., et al. Palladium(II)-catalyzed oxidative dearomatization of free naphthols with two alkyne units. *Org. Lett.* **16**, 6132–6135 (2014).
5. Liu, J. G. & Ueda, M. High refractive index polymers: fundamental research and practical applications. *J. Mater. Chem.* **19**, 8907–8919 (2009).
6. Ohkubo, K., Nanjo, T. & Fukuzumi, S. Efficient photocatalytic oxygenation of aromatic alkene to 1,2-dioxetane with oxygen via electron transfer. *Org. Lett.* **7**, 4265–4268 (2005).
7. Kwak, G., Fujiki, M., Sakaguchi, T. & Masuda, T. Mono- and multicolor FL image patterning based on highly luminous diphenylacetylene polymer derivative by facile photobleaching. *Macromolecules* **39**, 319–323 (2006).
8. Hu, R. R., et al. Facile synthesis of soluble nonlinear polymers with glycogen-like structures and functional properties from "simple" acrylic monomers. *Polym. Chem.* **4**, 95–105 (2013).
9. Wu, L. Q., Abada, Z., Lee, D. S., Poliakoff, M. & George, M. W. Combining engineering and chemistry for the selective continuous production of four different oxygenated compounds by photo-oxidation of cyclopentadiene using liquid and supercritical CO_2 as solvents. *Tetrahedron* **74**, 3107–3112 (2018).
10. Suzuki, M., et al. Photo-oxidation of 1,3-cyclopentadiene using partially quaternized poly(1-vinylimidazole)-bound ruthenium(II) complexes. *Phys. Chem. Chem. Phys.* **2**, 109–114 (2000).
