# Supplementary material for: Photoresponsive spiro-polymers generated in situ by C–H-activated polyspiroannulation
Source: Nat Commun. 2019 Dec 2;10:5483. doi: 10.1038/s41467-019-13308-w (PMC6889291; doi:10.1038/s41467-019-13308-w)
Supplement: Supplementary file 3 — Reporting Summary [file 41467_2019_13308_MOESM3_ESM.pdf]

Corresponding author(s): Ben Zhong Tang (tangbenz@ust.hk); Jacky W.Y. Lam (chjacky@ust.hk)

Lasing Reporting Summary

Nature Research wishes to improve the reproducibility of the work that we publish. This form is intended for publication with all accepted papers reporting claims of lasing and provides structure for consistency and transparency in reporting. Some list items might not apply to an individual manuscript, but all fields must be completed for clarity.

For further information on Nature Research policies, including our data availability policy, see Authors & Referees.

Experimental design

Please check: are the following details reported in the manuscript?

1. Threshold

Plots of device output power versus pump power over a wide range of values indicating a clear threshold

☐ Yes
☒ No

The laser that we used in this work is a commercially available laser (Santec TSL-510). This laser is not designed by us and it is just used as a measurement instrument in the photonic device experiments. The laser was used as received without any further modification. The brand and model of the laser was reported in the supplementary methods.

2. Linewidth narrowing

Plots of spectral power density for the emission at pump powers below, around, and above the lasing threshold, indicating a clear linewidth narrowing at threshold

☐ Yes
☒ No

The laser that we used in this work is a commercially available laser (Santec TSL-510). This laser is not designed by us and it is just used as a measurement instrument in the photonic device experiments. The laser was used as received without any further modification. The brand and model of the laser was reported in the supplementary methods.

Resolution of the spectrometer used to make spectral measurements

☐ Yes
☒ No

The laser that we used in this work is a commercially available laser (Santec TSL-510). This laser is not designed by us and it is just used as a measurement instrument in the photonic device experiments. The laser was used as received without any further modification. The brand and model of the laser was reported in the supplementary methods.

3. Coherent emission

Measurements of the coherence and/or polarization of the emission

☐ Yes
☒ No

The laser that we used in this work is a commercially available laser (Santec TSL-510). This laser is not designed by us. It is not the focus of this work but just used as a measurement instrument in the photonic device experiments. The laser was used as received without any further modification. Therefore, we did not measure the coherence and/or polarization of the emission. Instead, the brand and model of the laser was reported in the supplementary methods.

4. Beam spatial profile

Image and/or measurement of the spatial shape and profile of the emission, showing a well-defined beam above threshold

☐ Yes
☒ No

The laser that we used in this work is a commercially available laser (Santec TSL-510). This laser is not designed by us. It is not the focus of this work but just used as a measurement instrument in the photonic device experiments. The laser was used as received without any further modification. Therefore, we did not measure its spatial shape and profile of the emission. Instead, the brand and model of the laser was reported in the supplementary methods.

5. Operating conditions

Description of the laser and pumping conditions
Continuous-wave, pulsed, temperature of operation

☐ Yes
☒ No

The laser that we used in the photonic device experiments is a commercially available laser (Santec TSL-510). This laser is not designed by us and it is just used as a measurement instrument in this work. The laser was used as received without any further modification. The brand and model of the laser was reported in the supplementary methods and the temperature of operation was also provided.

Threshold values provided as density values (e.g. W cm^{-2} or J cm^{-2}) taking into account the area of the device

☐ Yes
☒ No

The laser that we used in this work is a commercially available laser (Santec TSL-510). This laser is not designed by us and it is just used as a measurement instrument in the photonic device experiments. The laser was used as received without any further modification. We did not test its threshold values. The brand and model of the laser was reported in the supplementary methods.

6. Alternative explanations

Reasoning as to why alternative explanations have been ruled out as responsible for the emission characteristics

e.g. amplified spontaneous, directional scattering; modification of fluorescence spectrum by the cavity

☐ Yes
☒ No

The laser that we used in this work is a commercially available laser (Santec TSL-510). This laser is not designed by us and it is just used as a measurement instrument in the photonic device experiments. The laser was used as received without any further modification. The brand and model of the laser was reported in the supplementary methods.

7. Theoretical analysis

Theoretical analysis that ensures that the experimental values measured are realistic and reasonable

e.g. laser threshold, linewidth, cavity gain-loss, efficiency

☐ Yes
☒ No

The laser that we used in the photonic device experiments is a commercially available laser (Santec TSL-510). This laser is not designed by us and it is just used as a measurement instrument in this work. The laser was used as received without any further modification. We did not do the theoretical analysis on it. The brand and model of the laser was reported in the supplementary methods.

8. Statistics

Number of devices fabricated and tested

☐ Yes
☒ No

The laser that we used in this work is a commercially available laser (Santec TSL-510). This laser is not designed by us and we did not fabricated and tested the related devices. It is just used as a measurement instrument in the photonic device experiments. The laser was used as received without any further modification. The brand and model of the laser was reported in the supplementary methods.

Statistical analysis of the device performance and lifetime (time to failure)

☐ Yes
☒ No

The laser that we used in this work is a commercially available laser (Santec TSL-510). This laser is not designed by us. It is just used as a measurement instrument in the photonic device experiments. The laser was used as received without any further modification. We did not do the statistical analysis of the device performance and lifetime. The brand and model of the laser was reported in the supplementary methods.
